# Alginate- and *κ*-carrageenan-supported asymmetric organocatalysts: preparation, characterization, and catalytic activity for Friedel–Crafts alkylation

**DOI:** 10.1039/d5ra05785j

**Published:** 2025-08-28

**Authors:** Manaho Murakishi, Shogo Nakanishi, Hiromitsu Sogawa, Fumio Sanda

**Affiliations:** a Department of Chemistry and Materials Engineering, Faculty of Chemistry, Materials and Bioengineering, Kansai University 3-3-35 Yamate-cho Suita Osaka 564-8680 Japan sogawa@kansai-u.ac.jp sanda@kansai-u.ac.jp

## Abstract

Alginate- and *κ*-carrageenan-supported imidazolidinone catalysts were newly prepared, and their catalytic activities for the Friedel–Crafts alkylation of 1-methylindole with crotonaldehyde were evaluated. The enantiomeric excess of the product was enhanced by using the polymer-supported catalysts consisting of an appropriate absolute configuration of imidazolidinones and alginate/*κ*-carrageenan *via* the cooperative effect that constructs a chiral environment more suited for asymmetric induction compared to imidazolidinone only. Further, the polymer-supported catalysts were easily removed from the reaction media by a simple filtration technique. *κ*-Carrageenan-supported catalysts were superior in terms of reusability compared to alginate-supported catalysts, likely due to the strong ionic interaction between the sulfate and ammonium moieties.

## Abstract 2

Alginate- and *κ*-carrageenan-supported imidazolidinone catalysts were newly prepared, and their catalytic activities for the Friedel–Crafts alkylation of 1-methylindole with crotonaldehyde were evaluated.

## Graphical abstract

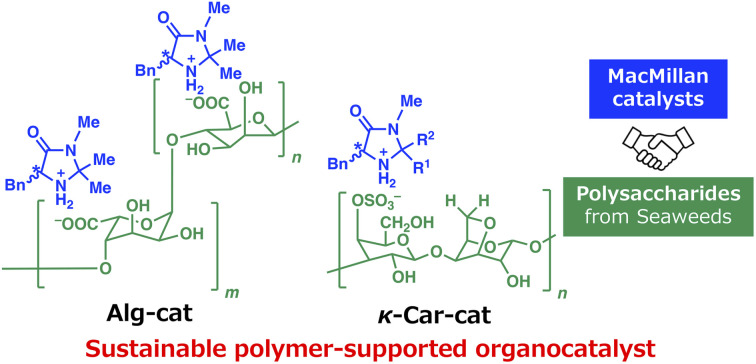

## Introduction

In recent years, efficient usage of biomass resources has attracted much attention in order to build a sustainable society. In particular, terrestrial biomass represented by cellulose is widely researched from basics to applications.^1^ Development of marine biomass-based materials is also essential and has been actively studied for a long period. For instance, chitin and chitosan, abundantly contained in crab shells, are applied in sensors and polymer-supported catalysts.^2^ Alginic acid (Alg) is a biocompatible polysaccharide contained in seaweed. Alg consists of two uronic acids, d-mannuronic acid and l-guluronic acid that are arranged in a linear and unbranched fashion. Alg sodium salt is soluble in water, whereas it turns into gel in the presence of multivalent cations such as Ca^2+^. Owing to these characteristics, Alg is applied in a wide variety of fields^3^ including pharmaceuticals,^4–6^ drug delivery systems,^7^ food packaging,^8^ cosmetics,^9^ water treatment materials,^10^ membranes,^11^ catalysts,^12^ hydrogels^13^ and adhesives.^14^ Meanwhile, carrageenan (Car) is a sulfated polysaccharide consisting of alternating d-galactose and 3,6-anhydro-d-galactose units linked by *α*-1,3-glycosidic and *β*-1,4-glycosidic bonds. There are about fifteen idealized carrageenan structures traditionally identified by Greek letters.^15^ They are defined by the number and position of sulphate groups, presence/absence of 3,6-anhydro-d-galactose, and conformation of the pyranoside ring.^16^*κ*-/*ι*-/*λ*-Cars are commercially available and mainly used in food, and also expected as precursors for green chemicals and fuels such as 5-hydroxymethyl furfural.^17^

Organocatalysts are actively studied in recent years because of their low toxicity and low environmental load compared to metal-containing catalysts. Chiral imidazolidinone derivatives developed by MacMillan, who won the Nobel Prize in Chemistry 2020, are one of the representative organocatalysts. Chiral imidazolidinones can be easily prepared from various *α*-amino acids, and catalyze various asymmetric transformations including asymmetric Diels–Alder reaction,^18^ 1,3-dipolar cycloaddition,^19^ Michael reaction^20^ and Friedel–Crafts alkylation.^21^ The catalytic mechanism involves the formation of an iminium intermediate by the reaction of secondary amine moiety of imidazolidinone with an electrophilic substrate, followed by further addition reactions.

Polymer-supported catalysts, in which catalytic sites are tethered to polymers by chemical bonding or physical adsorption, feature excellent recyclability and reusability because they are separatable and removable from reaction mixtures by simple filtration and/or precipitation processes, which are usually difficult for conventional catalysts. Alg and Car are often employed as organocatalysts by themselves.^12,22^ Alg and Car are also functionalized by loading catalytically active groups to afford useful polysaccharide-supported catalysts.^23–28^ For instance, Bernardi, Tanchoux and coworkers prepared Alg-supported organocatalysts by physically adsorbing cinchona alkaloids onto Alg-based gels, and applied them to asymmetric Michael addition.^28^ The catalysts were easily removed from the reaction media by filtration, and were reused for further runs although the catalytic activity decreased by repeating cycles due to leaching of cinchona alkaloids during recycling process. Meanwhile, Itsuno, Haraguchi and coworkers synthesized polyether-based catalysts bearing imidazolidinone moieties in the main chain to find they repeatedly catalyze asymmetric Diels–Alder reaction without lowering catalytic activity and selectivity.^29^ Price, Michaelis and coworkers developed enzyme-inspired bifunctional helical peptide-based catalyst tethering an imidazolidinone and a thiourea. The inserted two functional groups cooperatively acted by proximity effect to exhibit enhanced catalytic activity and selectivity in Diels–Alder reaction and indole alkylation.^30^ Although these polymer-supported imidazolidinone catalysts are definitely advantageous and beneficial, it still remains a challenging topic to prepare and reuse them by a simple and environmentally friendly approach and/or to improve their enantioselectivity and catalytic activity by appropriate molecular designs. Based on these backgrounds, in the present study, we prepared polymer-supported catalysts by immobilizing chiral imidazolidinones to sodium alginate (Alg-Na) and *κ*-Car, and evaluated their catalytic activities and recycling properties in the Friedel–Crafts reaction in order to fabricate novel marine-biomass based functional materials. As far as we know, the present study is the first example of Alg- and *κ*-Car-based chiral induction catalysts utilizing MacMillan catalysts.

## Results and discussion

### Characterization of Alg-supported catalysts

**Scheme 1:**
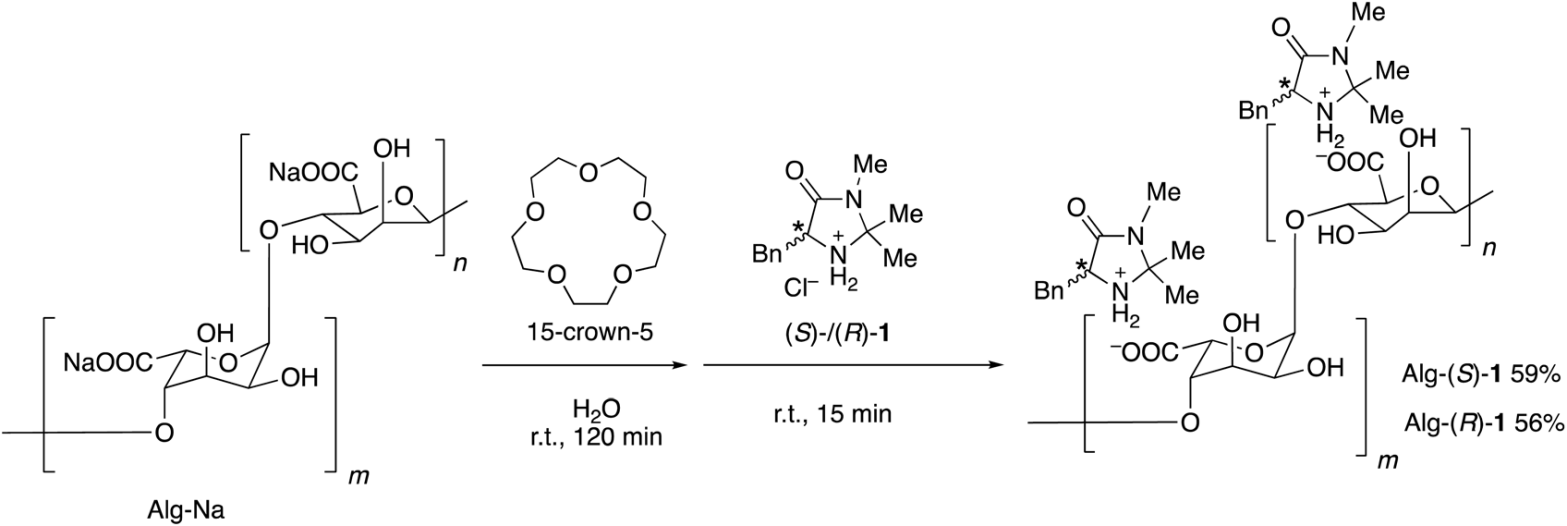
Preparation of Alg-(*S*)/(*R*)-1.

**Fig. 1:**
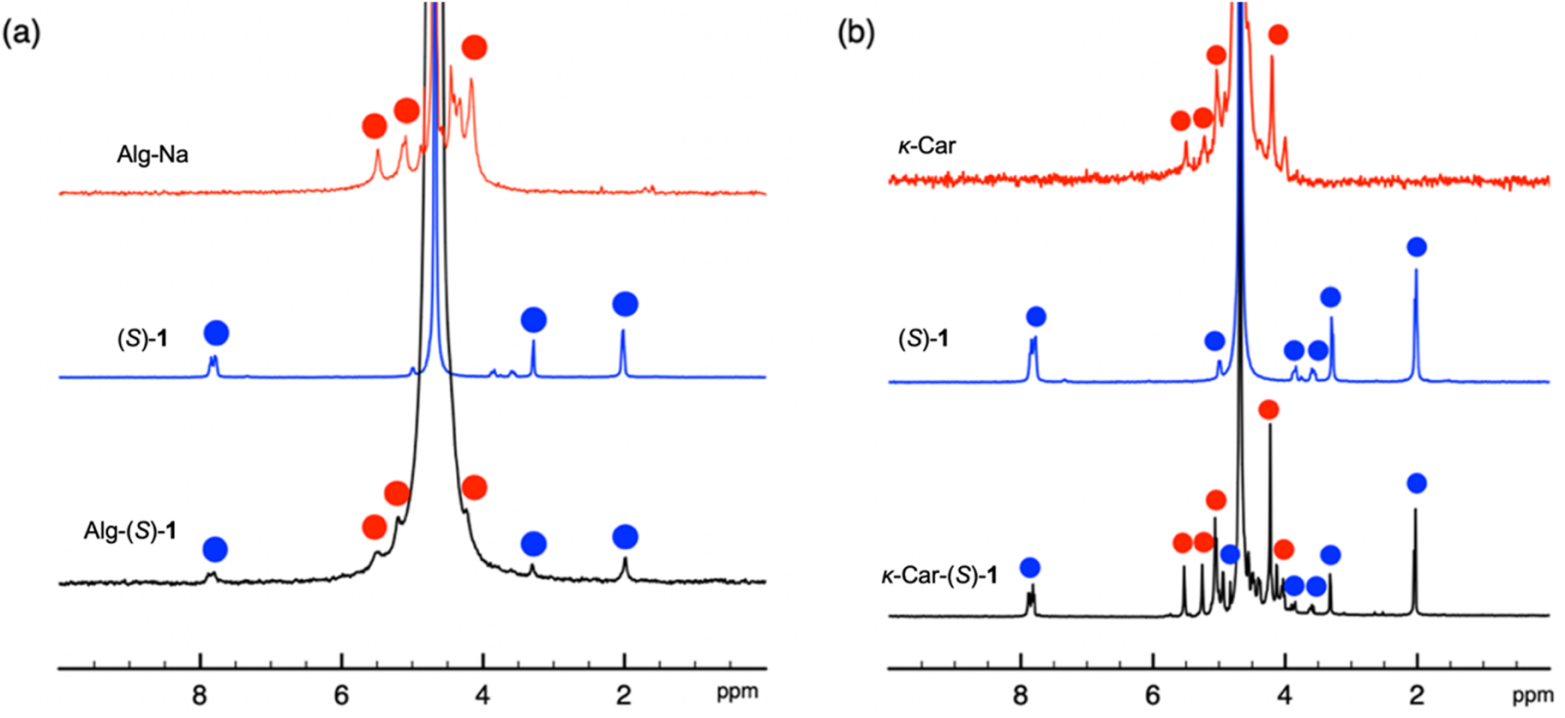
^1^H NMR (400 MHz) spectra of (a) Alg-Na, (*S*)-1 and Alg-(*S*)-1, and (b) *κ*-Car, (*S*)-1 and *κ*-Car-(*S*)-1 measured in D_2_O at 80 °C.

**Fig. 2:**
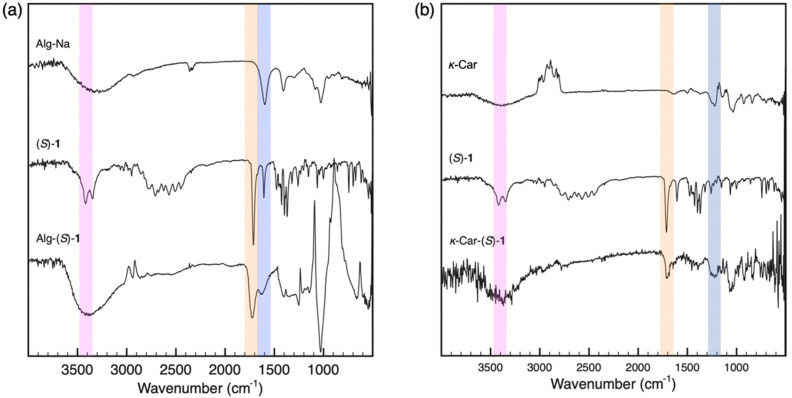
ATR-IR spectra of (a) Alg-Na, (*S*)-1 and Alg-(*S*)-1, and (b) *κ*-Car, (*S*)-1 and *κ*-Car-(*S*)-1.

Alg-(*S*)/(*R*)-1 were prepared by loading (*S*)/(*R*)-1 to Alg-Na according to Scheme 1. 15-Crown-5 was used to enhance the ion exchange according to the method by Iida and coworkers.^31^Fig. 1a shows the ^1^H NMR spectra of Alg-Na, (*S*)-1 and Alg-(*S*)-1. The NMR spectra were measured at 80 °C because the peaks of Alg-Na and Alg-(*S*)-1 were hardly detectable at room temperature. Alg-(*S*)-1 showed peaks originating from both Alg-Na and (*S*)-1, *e.g.*, broad peaks around at 4.0–6.0 ppm which are assignable to the main chain of Alg-Na, and a peak at 7.8 ppm assignable to the benzylic proton of (*S*)-1. Fig. 2a shows the IR spectra of Alg-Na, (*S*)-1 and Alg-(*S*)-1. Alg-Na showed peaks of O–H and C=O stretching vibration of Alg backbone at 3400 and 1600 cm^−1^, respectively. In addition, Alg-(*S*)-1 showed a C=O stretching vibration peak derived from (*S*)-1 at 1720 cm^−1^, indicating the successful preparation of Alg-(*S*)-1. Alg-(*R*)-1 showed ^1^H NMR and IR spectroscopic results similar to Alg-(*S*)-1 as shown in Fig. S1 and S11, respectively, which also indicate the successful preparation of Alg-supported catalysts in spite of the opposite absolute configuration of the imidazolidinone moiety. It was difficult to determine the incorporation ratio of imidazolidinone unit in the Alg-supported catalysts by ^1^H NMR spectroscopic measurement due to overlap with residual H_2_O/HDO proton signals, and by UV-vis diffuse reflectance and absorption spectroscopic measurements (Fig. S17 and S18) due to their low solubility and broad signals.

### Characterization of *κ*-Car-supported catalysts

**Scheme 2:**
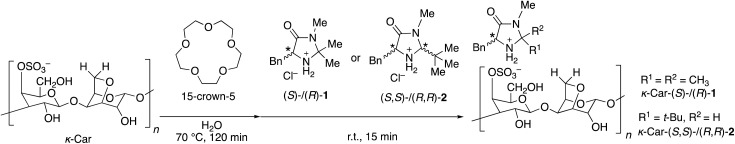
Preparation of *κ*-Car-(*S*)/(*R*)-1 and *κ*-Car-(*S*,*S*)/(*R*,*R*)-2. The cation species of *κ*-Car are unclear due to its natural origin.

**Fig. 3:**
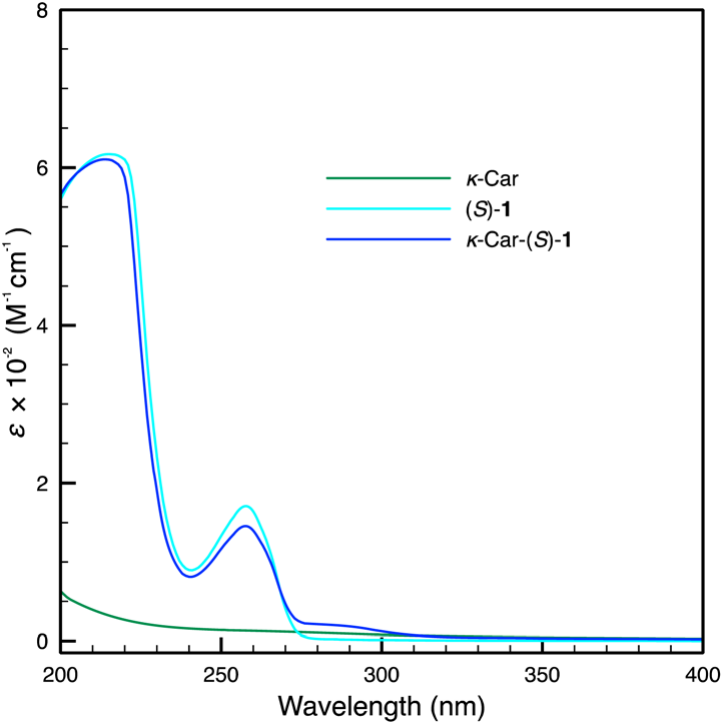
UV-vis absorption spectra of *κ*-Car, (*S*)-1 and *κ*-Car-(*S*)-1 measured in H_2_O (*c* = 5.0 mM).

*κ*-Car-supported catalysts, *κ*-Car-(*S*)/(*R*)-1 and *κ*-Car-(*S*,*S*)/(*R*,*R*)-2 were also prepared in a manner similar to Alg-(*S*)/(*R*)-1 according to Scheme 2. Although it has been reported that (*S*)/(*R*)-1 exhibits lower enantioselectivity than (*S*,*S*)/(*R*,*R*)-2,^21^ (*S*)/(*R*)-1 is less expensive and more readily available than (*S*,*S*)/(*R*,*R*)-2. Thus, large-scale experiments were more feasible. For these reasons, the catalysts were synthesized using (*S*)/(*R*)-1 to examine the catalytic reaction as well as the experiments using (*S*,*S*)/(*R*,*R*)-2 in the present study. Fig. 1b shows the ^1^H NMR spectra of *κ*-Car, (*S*)-1 and *κ*-Car-(*S*)-1 measured at 80 °C. *κ*-Car-(*S*)-1 showed peaks assignable to both *κ*-Car and (*S*)-1. The ^1^H NMR spectroscopic peaks were sharp compared with the Alg-derivatives, presumably owing to the better solubility in D_2_O. Based on the integral ratio between the peaks at 5.5 ppm (*κ*-Car) and 7.8 ppm ((*S*)-1), the incorporation ratio of (*S*)-1 in *κ*-Car-(*S*)-1 was estimated at 86%. Fig. 2b shows the IR spectra of *κ*-Car, (*S*)-1, and *κ*-Car-(*S*)-1. *κ*-Car-(*S*)-1 showed peaks of O–H and S=O stretching vibration of *κ*-Car at 3400 cm^−1^ and 1220 cm^−1^, respectively. In addition, *κ*-Car-(*S*)-1 showed a C=O stretching vibration peak derived from (*S*)-1 at 1700 cm^−1^. *κ*-Car-(*R*)-1 (Fig. S2 and S12) and *κ*-Car-(*S*,*S*)-/(*R*,*R*)-2 also showed reasonable ^1^H NMR and IR spectroscopic patterns (Fig. S3, S4, S13 and S14). These results strongly supported the successful preparation of a series of *κ*-Car-supported catalysts. Meanwhile, the incorporation ratio of (*S*)-1 in *κ*-Car-(*S*)-1 was also analyzed by UV-vis absorption spectra (Fig. 3). *κ*-Car showed no significant peak at 200–400 nm, whereas *κ*-Car-(*S*)-1 showed a peak around 258 nm assignable to the benzene ring of (*S*)-1. Based on the calibration curve prepared by measuring (*S*)-1 at various concentrations, the incorporation ratio of (*S*)-1 was estimated at 85%, which was almost consistent with that determined by ^1^H NMR spectroscopy. The incorporation ratios of *κ*-Car-(*R*)-1 and *κ*-Car-(*S*,*S*)/(*R*,*R*)-2 were also estimated in a similar manner as follows; *κ*-Car-(*R*)-1: 121% (NMR), 83% (UV-vis); *κ*-Car-(*S*,*S*)-2: 64% (NMR), 59% (UV-vis); *κ*-Car-(*R*,*R*)-2: 25% (NMR), 79% (UV-vis). In the present study, the UV-vis-determined incorporation ratios were applied for calculating the catalyst amount because ^1^H NMR peaks were broadened in some cases, possibly accompanied experimental errors larger than UV-vis-determination. An excess amount (typically 10 equiv.) of (*S*)-1 was added to –OSO_3_^−^ of *κ*-Car to achieve a high incorporation ratio. When *κ*-Car-(*S*)-1 was prepared by reducing the feed ratio of (*S*)-1 to 5 and 3 equiv., the incorporation ratios of (*S*)-1 were estimated at 49% and 20% based on ^1^H NMR spectroscopic measurements (Fig. S5 and S6), and at 41 and 29% based on UV-vis absorption spectroscopic measurements (Fig. S19). Thus, *κ*-Car-(*S*)-1, obtained with 10 equiv. of (*S*)-1, was used for asymmetric Friedel–Crafts reaction described in the next section. *κ*-Car-(*S*)-1 was also prepared without adding 15-crown-5 to find the incorporation ratio was (NMR: 22%, UV-vis: 52%) (Fig. S7 and S20). Although the cation species of *κ*-Car are unclear due to its natural origin, 15-crown-5 enhanced the ion exchange to give *κ*-Car-(*S*)-1 at a higher incorporation ratio of (*S*)-1 as expected.^31^

### Asymmetric Friedel–Crafts alkylation using Alg-supported catalysts

**Scheme 3:**
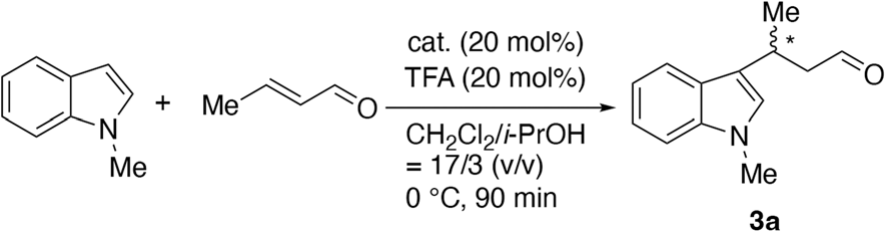
Asymmetric Friedel–Crafts alkylation of 1-methylindole with crotonaldehyde.

The Friedel–Crafts alkylation of 1-methylindole with crotonaldehyde was carried out using (*S*)/(*R*)-1 and Alg-(*S*)/(*R*)-1 as catalysts. The catalyst amount and reaction temperature were fixed at 20 mol% and 0 °C as the standard conditions (Scheme 3). Table 1 summarizes the product yield and enantiomeric excess (*ee*) data, which were determined by high performance liquid chromatography (HPLC) using a DAICEL CHIRALPAK AD-3, after converting the formed aldehyde 3a to the corresponding alcohol according to the literature.^28^ The yields exceeded 90% in most cases. Alg-(*S*)-1 gave a product with 21% *ee*, 15% lower than (*S*)-1 did (entries 1 and 3), while Alg-(*R*)-1 gave a product with 42% *ee*, 7% higher than (*R*)-1 did (entries 2 and 4). These results suggest that the chirality on the Alg and imidazolidinone moieties competed negatively in the former case, while the chirality cooperated to construct more suited chiral environment, improving the product *ee* in the latter case. Alg-(*R*)-1 did not give 3a without cocatalyst TFA (entry 5), whereas Alg-Na with TFA (entry 8) and TFA only (entry 9) systems gave a product almost quantitatively but with 0% *ee*. Based on these results, it is concluded that TFA partially catalyzed the reaction. It is also concluded that the chiral imidazoline unit played an important role in inducing the chirality of the product, while the Alg backbone alone is insufficient.

**Table 1:** Friedel–Crafts alkylation of 1-methylindole with crotonaldehyde using (*S*)/(*R*)-1 and Alg-supported catalysts^a^

Entry	Cat.	Yield^b^ (%)	*ee* c (%)
1	(*S*)-1	99 (94^d^)	36 (*R*)
2	(*R*)-1	98 (88^d^)	35 (*S*)
3	Alg-(*S*)-1	89	21 (*R*)
4	Alg-(*R*)-1	99	42 (*S*)
5	Alg-(*R*)-1^e^	Trace	—f
6	Alg-(*R*)-1_2nd_^g^	96	17 (*S*)
7	Alg-(*R*)-1^h^	91	10 (*S*)
8	Alg-Na	95	0
9	—i	83	0
10	—j	Trace	—f

a Conditions: [1-methylindole]_0_ = 0.5 M, [croton aldehyde]_0_ = 1.5 M, [cat.] = [trifluoroacetic acid (TFA)] = 0.1 M in CH_2_Cl_2_/*i*-PrOH = 17/3 (v/v) at 0 °C for 90 min. The catalytic amount was 20 mol% *vs.* 1-methylindole.

b Determined by ^1^H NMR.

c Determined by HPLC using a DAICEL CHIRALPAK AD-3 eluted with hexane/EtOH = 98/2 (v/v) at a flow rate of 1.0 mL min^−1^.

d Isolated yield.

e Without TFA.

f Not measured.

g Alg-(*R*)-1 was recovered from a reaction mixture, and used again.

h [cat.] = 0.05 M, the catalytic amount was reduced from 20 mol% to 10 mol% *vs.* 1-methylindole.

i Without catalyst but with TFA, 20 mol% *vs.* 1-methylindole.

j Without catalyst and TFA.

Next, recyclability of Alg-(*R*)-1 was examined. The catalyst was easily recovered and separated by filtration after the reaction. The recovered catalyst, Alg-(*R*)-1_2nd_ gave 3a almost quantitatively (entry 6), but the *ee* was significantly low (17%) compared with Alg-(*R*)-1 (entry 4), presumably because (*R*)-1 was partly leached away during the recycling process. This assumption is supported by the result that the yield was kept more than 90% with lowering a product ee to 10% by reducing the catalyst amount from 20 to 10 mol% (entries 4 and 7). We tried to analyze the conformation of Alg-(*S*)-/(*R*)-1 more precisely by NMR and circular dichroism (CD) spectroscopic measurements, but failed to obtain satisfactory data because Alg-(*S*)-/(*R*)-1 were hardly soluble in various solvents including H_2_O. Therefore, we focused on *κ*-Car-supported catalysts because they are completely soluble in H_2_O, and thus, the more detailed characterization is possible. Additionally, *κ*-Car-supported catalysts are expected to interact more strongly with MacMillan catalyst because of stronger ionic interaction between –OSO_3_^−^ group and ammonium moiety.

### Asymmetric Friedel–Crafts alkylation using *κ*-Car-supported catalysts

In a similar manner to Alg-supported catalysts, the Friedel–Crafts alkylation of 1-methylindole with crotonaldehyde was conducted using *κ*-Car-(*S*)/(*R*)-1 and *κ*-Car-(*S*,*S*)/(*R*,*R*)-2 as catalysts. As summarized in Table 2, *κ*-Car-(*S*)/(*R*)-1 showed a similar trend with Alg-(*S*)/(*R*)-1. Namely, *κ*-Car-(*S*)-1 gave a product 3a with 31% *ee*, 5% lower than (*S*)-1 (entries 1 and 3), whereas *κ*-Car-(*R*)-1 gave 3a with 40% *ee*, 5% higher than (*R*)-1 (entries 2 and 4). The chirality on the *κ*-Car and imidazolidinone moieties acted competitively in the former case, while acted cooperatively in the latter case. The CD and UV-vis absorption spectroscopic analysis, along with density functional theory calculations might support these competitive and cooperative effects, which are described in detail in the SI. Compared with (*S*)/(*R*)-1, (*S*,*S*)-/(*R*,*R*)-2 exhibited higher enantioselectivity due to the more exposed *Re* or *Si* face of the formed iminium 1, resulting in the formation of more favorable intermediate for new carbon–carbon bond formation.^32^ As expected, *κ*-Car-(*S*,*S*)-2 and *κ*-Car-(*R*,*R*)-2 gave a product with higher ee compared to *κ*-Car-(*S*)-1 and *κ*-Car-(*R*)-1 (entries 3, 4, 7 and 8). On the contrary, *κ*-Car-(*S*,*S*)-2 and *κ*-Car-(*R*,*R*)-2 did not exhibit chirality-competitive and cooperative effects compared with (*S*,*S*)-2 and (*R*,*R*)-2 (entries 5–8), differently from *κ*-Car-(*S*)/(*R*)-1 mentioned above. It should be noted that the polymer-supported catalysts showed higher catalytic activity *via* immobilization of (*S*,*S*)/(*R*,*R*)-2. The chirality on *κ*-Car apparently enhanced the product *ee* although *κ*-Car itself showed no enantioselectivity (entry 11). Next, catalyst loading amount was changed in a range of 5–20 mol% using *κ*-Car-(*R*,*R*)-2. As shown in Table S1, the catalytic activity and product *ee* were maintained even reducing the catalyst and TFA contents from 20 mol% to 5 mol%. As (*R*,*R*)-2 exhibited a similar trend, this high catalytic activity was not endowed by the immobilization to *κ*-Car. However, it should be noted that such a small catalytic amount is enough to give a product with a high *ee*. The temperature dependency was also evaluated as summarized in Table S2. The *ee* slightly decreased by raising temperature from 0 to 25 °C, while did not increase by lowering temperature from 0 °C to −50 °C.

**Table 2:** Friedel–Crafts alkylation of 1-methylindole with crotonaldehyde using (*S*)/(*R*)-1, (*S*,*S*)-/(*R*,*R*)-2 and *κ*-Car-supported catalysts^a^

Entry	Cat.	Yield^b^ (%)	*ee* c (%)
1	(*S*)-1	99 (94^d^)	36 (*R*)
2	(*R*)-1	98 (87^d^)	35 (*S*)
3	*κ*-Car-(*S*)-1	96	31 (*R*)
4	*κ*-Car-(*R*)-1	99	40 (*S*)
5	(*S*,*S*)-2	95	79 (*R*)
6	(*R*,*R*)-2	96	78 (*S*)
7	*κ*-Car-(*S*,*S*)-2	99	86 (*R*)
8	*κ*-Car-(*R*,*R*)-2	99	85 (*S*)
9	*κ*-Car-(*R*,*R*)-2_2nd_	99	84 (*S*)
10	*κ*-Car-(*R*,*R*)-2_3rd_	96	67 (*S*)
11	*κ*-Car	98	0

a Conditions: [1-methylindole]_0_ = 0.5 M, [croton aldehyde]_0_ = 1.5 M, [cat.] = [trifluoroacetic acid (TFA)] = 0.1 M in CH_2_Cl_2_/*i*-PrOH = 17/3 (v/v) at 0 °C for 90 min. The catalytic amount was 20 mol% *vs.* 1-methylindole.

b Determined by ^1^H NMR.

c Determined by HPLC using a DAICEL CHIRALPAK AD-3 eluted with hexane/EtOH = 98/2 (v/v) at a flow rate of 1.0 mL min^−1^.

d Isolated yield.

Next, the recyclability of *κ*-Car-(*R*,*R*)-2 was examined (Table 2, entries 8–10). The supernatant containing 3a was removed from the reaction vessel after the reaction by decantation, and the remaining *κ*-Car-(*R*,*R*)-2 was washed three times with CH_2_Cl_2_/*i*-PrOH = 85/15 (v/v) and vacuum dried before reuse. Then, the recovered *κ*-Car-(*R*,*R*)-2 and 20 mol% TFA were fed in the next catalytic cycle under the same reaction conditions. *κ*-Car-(*R*,*R*)-2 in the second cycle gave a product with almost the same yield and *ee* (entry 9) as those in the first cycle (entry 8). Thus, the recyclability was remarkably improved compared to Alg-(*R*)-1 likely due to the stronger ionic interaction between the –OSO_3_^−^ group of *κ*-Car and ammonium moiety of (*R*,*R*)-2. The product yield was almost maintained in the third cycle, but the ee decreased to 67% (entry 10), probably due to the leaching of (*R*,*R*)-2 from *κ*-Car. To evaluate the amount of remaining (*R*,*R*)-2 in *κ*-Car-(*R*,*R*)-2 after the third cycle, the UV-vis absorption spectra were measured. As shown in Fig. S21, *κ*-Car-(*R*,*R*)-2 broadly absorbed at all the measurement range from 240 to 360 nm, differently from *κ*-Car and (*R*,*R*)-2. Judging from the UV-vis absorption of 3a in the Friedel–Crafts reaction, *κ*-Car-(*R*,*R*)-2 seems to adsorb 3a, decreasing the chiral catalytic activity. Although it was difficult to estimate remaining (*R*,*R*)-2 in the *κ*-Car-(*R*,*R*)-2 precisely, an undesirable change of the catalyst was confirmed by UV-vis absorption spectroscopic measurements.

**Scheme 4:**
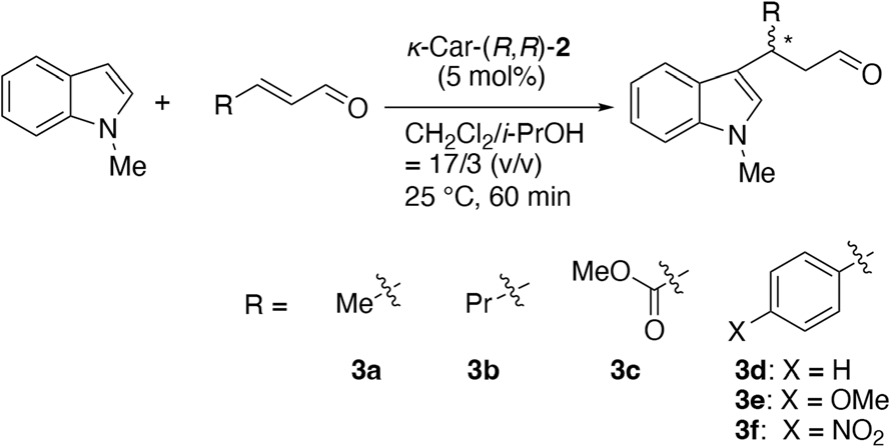
Asymmetric Friedel–Crafts alkylation of 1-methylindole with various aldehydes.

Finally, we investigated the substrate scope of aldehydes using *κ*-Car-(*R*,*R*)-2. In addition to crotonaldehyde, 1-hexanal, methyl fumaraldehydate and cinnamaldehyde derivatives as representative aromatic aldehydes were employed (Scheme 4). Considering the previously optimized conditions, a low catalyst loading (5 mol%) was employed. Further, since the reaction proceeded even at 5 mol% TFA content, the reactions were carried out even without TFA at this time. As will be discussed later, the reactions of cinnamaldehyde derivatives gave the products in low yields. Therefore, although a slight decrease in enantioselectivity was predicted, the reactions were carried out at 25 °C instead of 0 °C. As summarized in Table 3, *κ*-Car-(*R*,*R*)-2 gave product 3a with 70% *ee* even under TFA-free conditions at 25 °C (entry 1). Similarly, 3b bearing a long alkyl chain was obtained with 68% *ee* (entry 2), comparable to that of 3a. In the case of 3c, both the yield and ee decreased, affording the (*R*)-enantiomer with 34% *ee* (entry 3). The absolute configurations of 3b and 3c were determined according to the literature,^33^ in which enantiomerically opposite 3c was obtained compared to 3a and 3b. On the other hand, cinnamaldehyde derivatives gave products 3d, 3e and 3f in trace yields (entries 4, 5 and 6). Especially, for methoxy-substituted cinnamaldehyde, the starting material was recovered unchanged, indicating no reaction occurred. These results indicate that *κ*-Car-(*R*,*R*)-2 effectively catalyzes the enantioselective reactions of aliphatic aldehydes, while does not toward aromatic aldehydes.

**Table 3:** Friedel–Crafts alkylation of 1-methylindole with various aldehydes using *κ*-Car-(*R*,*R*)-2^a^

Entry	Product	Yield^b^ (%)	*ee* c (%)
1	3a	99	70 (S)
2	3b	98	68 (*S*)
3	3c	92	34 (*R*)
4	3d	Trace	—d
5	3e	Trace	—d
6	3f	Trace	—d

a Conditions: [1-methylindole]_0_ = 0.5 M, [aldehyde]_0_ = 1.5 M, [κ-Car-(*R*,*R*)-2] = 25 mM in CH_2_Cl_2_/i-PrOH = 17/3 (v/v) at 25 °C for 60 min. The catalytic amount was 5 mol% *vs.* 1-methylindole.

b Determined by ^1^H NMR.

c Determined by HPLC using a DAICEL CHIRALPAK AD-3 eluted with hexane/EtOH = 98/2 (v/v) at a flow rate of 1.0 mL min^−1^.

d Not measured.

**Scheme 5:**
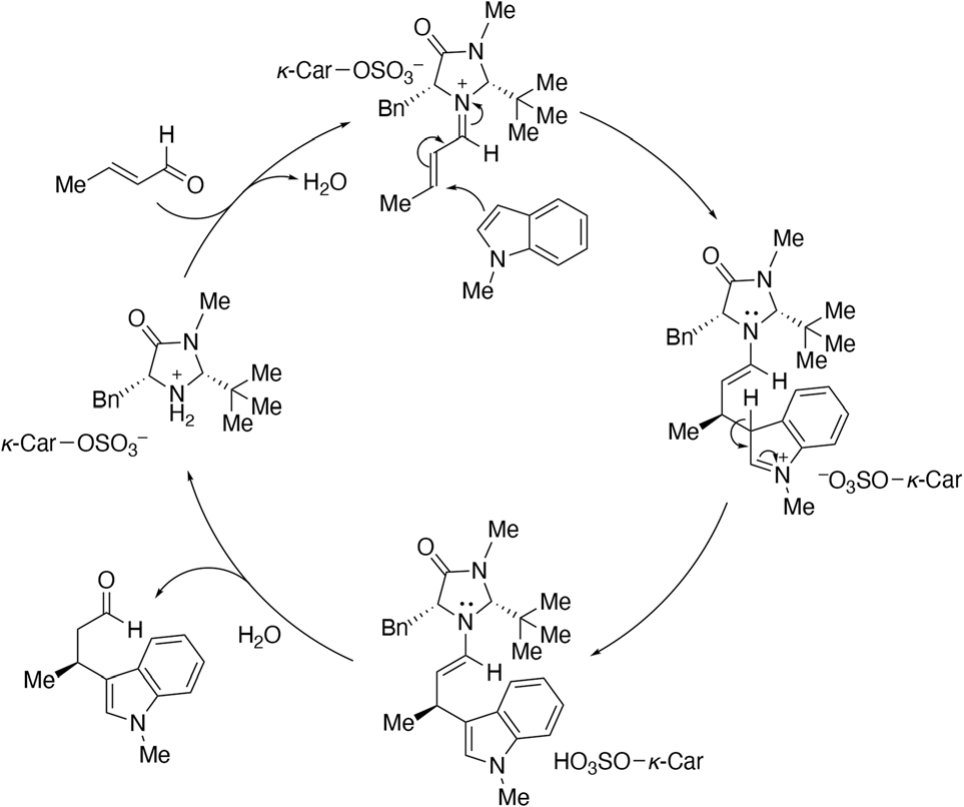
Plausible mechanism of asymmetric Friedel–Crafts alkylation catalyzed with *κ*-Car-(*R*,*R*)-2.

Scheme 5 illustrates the plausible mechanism of asymmetric Friedel–Crafts alkylation catalyzed with *κ*-Car-(*R*,*R*)-2. Initially, a highly electrophilic iminium intermediate is formed upon addition of the aldehyde to the ammonium moiety of imidazolidinone, which ionically interacts with –OSO_3_^−^ group in *κ*-Car-(*R*,*R*)-2. 1-Methylindole then nucleophilically attacks this intermediate to form the adduct. During the reaction, *κ*-Car remains in close proximity to the imidazolidinone reactive site *via* ionic interactions, contributing to the improvement of *ee*. The enamine intermediate partly loses the ionic interactions resulting in partial leaching of the imidazolidinone moiety from *κ*-Car. This detachment and the adsorption of the product are presumably responsible for the decrease in ee during the catalyst recycling process, as described above.

## Conclusions

Alg- and *κ*-Car-supported imidazolidinone catalysts were newly prepared and their catalytic activities for Friedel–Crafts alkylation were evaluated. By choosing absolute configurations of imidazolidinones and Alg/*κ*-Car appropriately, the chirality induction was enhanced by cooperative effect that constructs a chiral environment more suited for asymmetric induction. The reusability of the *κ*-Car-supported catalyst was superior to the Alg-supported counterpart likely due to the stronger ionic interaction between –OSO_3_^−^ and ammonium moieties than that between –CO_2_^−^ and ammonium moieties. The obtained results surely contribute for the development of sustainable functional materials based on marine biomass. Further expansion of substrates and reactions is ongoing.

## Author contributions

Manaho Murakishi: investigation; resources; writing – original draft. Shogo Nakanishi: investigation. Hiromitsu Sogawa: conceptualization; funding acquisition; resources; supervision; writing – review & editing. Fumio Sanda: supervision; writing – review & editing.

## Conflicts of interest

The authors declare no competing financial interest.

## Supplementary Material

RA-015-D5RA05785J-s001

RA-015-D5RA05785J-s002

## Data Availability

The data supporting this article have been included as part of the SI. Experimental section; ^1^H NMR spectra of the catalysts and products (Fig. S1–S10), ATR-IR spectra of the catalysts (Fig. S11–S16), UV-vis absorption spectra of the Alg- and *κ*-Car-supported catalysts (Fig. S17–S21), CD and UV-vis absorption spectra of *κ*-Car-supported catalysts (Fig. S22 and S23), simulated CD and UV-vis absorption spectra (Fig. S24), effects of catalyst and cocatalyst loading (Table S1) and temperature (Table S2) on the Friedel–Crafts alkylation, HPLC charts of the products (Fig. S25–S39). See DOI: https://doi.org/10.1039/d5ra05785j.
