# Supplementary material for: Alginate- and κ-carrageenan-supported asymmetric organocatalysts: preparation, characterization, and catalytic activity for Friedel–Crafts alkylation
Source: RSC Adv. 2025 Aug 28;15(37):30865–71. doi: 10.1039/d5ra05785j (PMC12395031; doi:10.1039/d5ra05785j)
Supplement: RA-015-D5RA05785J-s002 [file RA-015-D5RA05785J-s002.pdf]

Supplementary Information (SI) for RSC Advances.
This journal is © The Royal Society of Chemistry 2025

Supporting Information

Alginate- and κ-carrageenan-supported asymmetric organocatalysts: preparation, characterization, and catalytic activity for Friedel–Crafts alkylation

Manaho Murakishi,[a] Shogo Nakanishi,[a] Hiromitsu Sogawa,*,[a] Fumio Sanda*,[a]

[a]*Department of Chemistry and Materials Engineering, Faculty of Chemistry, Materials and Bioengineering, Kansai University, 3-3-35 Yamate-cho, Suita, Osaka 564-8680, Japan*

* Corresponding Authors. E-mail: sogawa@kansai-u.ac.jp, sanda@kansai-u.ac.jp

EXPERIMENTAL

Materials

Alginate (Alg-Na, 300 cps) and crotonaldehyde were purchased from Nacalai tesque Inc. (Kyoto, Japan). (*S*)-(-)-5-Benzyl-2,2,3-trimethyl-4-imidazolidinone hydrochloride, (*R*)-(+)-5-benzyl-2,2,3-trimethyl-4-imidazolidinone hydrochloride, (2*S*,5*S*)-(-)-2-*tert*-butyl-3-methyl-5-benzyl-4-imidazolidinone, (2*R*,5*R*)-(+)-2-*tert*-butyl-3-methyl-5-benzyl-4-imidazolidinone, 15-crown-5-ether, sodium borohydride, and *κ*-carrageenan (*κ*-Car) were purchased from Tokyo Chemical Industry Co., Ltd (Tokyo, Japan). 1-Methylindole was purchased from Sigma Aldrich Japan (Tokyo, Japan). All the other reagents and solvents were commercially obtained and used as received without purification.

Measurements

^{1}H (400 MHz) nuclear magnetic resonance (NMR) spectra were recorded on a JEOL JNM–ECZ400 and a JEOL JNM–ECS400 spectrometers. IR spectra were measured on a JASCO IR–4100 spectrophotometer. For attenuated total reflection (ATR) measurements, ATR PRO410–S using a Ge prism was equipped. UV–vis absorption spectra were measured in a quartz cell (optical path length: 1 cm) using a JASCO V–500 spectrophotometer. Diffuse reflection UV–vis spectra were measured on a Shimazu UV-

visible spectrophotometer UV–2600. The enantiomeric ratio was determined by chiral HPLC (JASCO LC–NetII/ADC, JASCO UV–4070, JASCO PU–0580 plus, DAICEL CHIRALPAK AD-3, eluent: hexane/EtOH = 98/2 (v/v)). CD spectra were measured in a quartz cell (optical path length 1 cm) using a JASCO J–800 spectrophotometer.

Preparation of alginate-supported catalysts

The polymer-supported catalysts, Alg-(*S*)/(*R*)-**1** were prepared according to Scheme 1, referring reaction conditions of the literature.[S1)]

Alg-(*S*)-**1**

15-Crown-5-ether (0.196 mL, 0.990 mmol) was added to an aqueous solution of Alg-Na (4.00 mL, 85 mM based on a repeating unit, 0.340 mmol), and the resulting mixture was stirred at room temperature for 2 hours. The resulted aqueous solution was washed with Et_2O (50 mL) to remove unreacted 15-crown-5-ether, and lyophilized to give a white powder. To the obtained solid, water (4.4 mL) was added to prepare a 78 mM aqueous solution. In the next step, (*S*)-**1** (866 mg, 3.40 mmol) was added, and the mixture was stirred for 15 min at room temperature. The formed complex was precipitated in EtOH (100 mL), collected by filtration, washed with EtOH, and dried in vacuo to give Alg-(*S*)-

1 (86.2 mg). Yield: 59%. ^{1}H NMR (400 MHz, D_2O, 80 °C): δ 8.24–7.69 (m, 5H), 5.55 (s, 1H), 5.21 (s, 1H), 4.27–4.13 (3H), 3.30 (s, 3H), 1.99 (s, 6H). IR (ATR): 3370, 1718, 1636, 1398, 1250, 1213, 1144, 1028, 925, 662, 578, 566, 547, 501 cm^{-1}.

Alg-(*R*)-**1**

Alg-(*R*)-**1** was prepared in a similar manner to Alg-(*S*)-**1** using (*R*)-**1** (866 mg, 3.40 mmol). Yield: 56%. ^{1}H NMR (400 MHz, D_2O, 80 °C): δ 7.89–7.82 (m, 5H), 5.52 (d, J = 32.0 Hz, 2H), 5.22 (s, 2H), 4.49 (d, J = 8.2 Hz, 3H), 3.88 (s, 1H), 3.31 (s, 3H), 2.01 (s, 6H), 1.62 (t, J = 3.4 Hz, 17H). IR (ATR): 3443, 3419, 2948, 1718, 1633, 1398, 1368, 1250, 1030, 809, 753, 671, 609, 572 cm^{-1}

Preparation of *κ*-Car-supported catalysts

The polymer-supported catalysts, *κ*-Car-(*S*)/(*R*)-**1**, and *κ*-Car-(*S*,*S*)-/(*R*,*R*)-**2** were prepared according to Scheme 2. Commercially available (2*S*,5*S*)-(-)-2-*tert*-butyl-3-methyl-5-benzyl-4-imidazolidinone and (2*R*,5*R*)-(+)-2-*tert*-butyl-3-methyl-5-benzyl-4-imidazolidinone were treated with 4 M HCl aq. to form hydrochloride salts (*S*,*S*)-**2** and (*R*,*R*)-**2** before the reaction.

κ-Car-(*S*)-**1**

15-Crown-5-ether (0.196 mL, 0.990 mmol) was added to an aqueous solution of *κ*-Car (16.0 mL, 21.3 mM, 0.340 mmol) in a 10 mL eggplant flask, and the resulting mixture was stirred for 2 h at 70 °C. The resulted aqueous solution was washed with Et_2O (50 mL), and lyophilized to give a white powder (520 mg). To the obtained solid, water (4.4 mL) was added to prepare a 78 mM aqueous solution. In the next step, (*S*)-**1** (866 mg, 3.40 mmol) was added, and the mixture was stirred at room temperature for 15 min. The obtained polymer complex was precipitated in EtOH (100 mL), collected by membrane filtration, washed with EtOH, and dried in vacuo to give *κ*-Car-(*S*)-**1** (252 mg). Yield: quantitative. ^{1}H NMR (400 MHz, D_2O, 80 °C) δ 7.89–7.79 (m, 5H), 5.52 (s, 1H), 5.25 (s, 1H), 5.05 (d, J = 7.8 Hz, 3H), 4.96–4.93 (m, 2H), 4.40 (s, 1H), 4.22 (s, 3H), 4.13 (d, J = 5.0 Hz, 3H), 4.03 (t, J = 8.2 Hz, 1H), 3.88–3.55 (m, 2H), 3.31 (d, J = 4.6 Hz, 3H), 2.01 (d, J = 5.0 Hz, 6H). IR (ATR): 3450, 3416, 1714, 1227, 1070, 611 cm^{-1}.

κ-Car-(*S*)-**1** without adding 15-crown-5-ether

To elucidate the effect of 15-crown-5-ether, (*S*)-**1** was directly added to *κ*-Car as shown in Scheme S3. The detailed experimental procedure is as follows.

(*S*)-**1** (255 mg, 1.00 mmol) was added to an aqueous solution of *κ*-Car (8.80 mL, 11.4 mM, 0.100 mmol) in a 10 mL eggplant flask, and the mixture was stirred at 70 °C for 15 min. The obtained polymer complex was precipitated in EtOH (80 mL), collected by membrane filtration, washed with EtOH, and dried in vacuo to give *κ*-Car-(*S*)-**1** (43.3 mg). Yield: 72%.

Scheme S3. Preparation of *κ*-Car-(*S*)-**1** without 15-crown 5

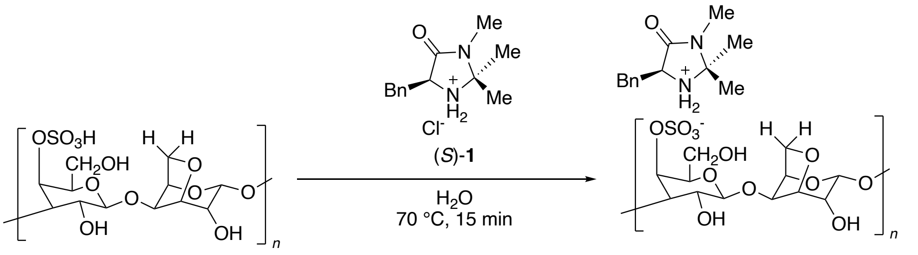

<u>*κ*-Car-(*R*)-**1**</u>

κ-Car-(*R*)-**1** was prepared in a similar manner to *κ*-Car-(*S*)-**1** using (*R*)-**1** (866 mg, 3.40 mmol). Yield: 94%. ^{1}H NMR (400 MHz, D_2O, 80 °C) *δ* 7.86–7.77 (m, 5H), 5.50 (s, 1H), 5.23 (s, 1H), 5.03 (d, *J* = 10.9 Hz, 1H), 4.92–4.89 (m, 0H), 4.54 (s, 1H), 4.37 (d, *J* = 10.4 Hz, 1H), 4.21 (s, 2H), 4.10 (d, *J* = 3.2 Hz, 4H), 4.02 (d, *J* = 9.5 Hz, 0H), 3.85–3.52 (m, 2H), 3.28 (d, *J* = 2.7 Hz, 3H), 1.97 (t, *J* = 3.4 Hz, 6H). IR (ATR): 3378, 2939, 1699, 1428, 1396, 1250, 1212, 1157, 922, 842, 733, 700, 547, 516, 504 cm^{-1}.

κ- Car-(*S*,*S*)-**2**

κ-Car-(*S*,*S*)-**2** was prepared in a similar manner to *κ*-Car-(*S*)-**1** using (*S*,*S*)-**2** (962 mg, 3.40 mmol). Yield: 79%. ^{1}H NMR (400 MHz, D_2O) δ 7.89 (s, 5H), 5.54 (s, 4H), 5.27 (s, 4H), 5.07 (s, 7H), 4.42 (s, 2H), 4.24 (s, 9H), 4.15 (s, 4H), 3.59–3.68 (1H), 3.06–3.16 (2H), 1.62 (s, 9H). IR (ATR): 3391, 3354, 2903, 1716, 1508, 1252, 1037, 927, 843, 507 cm^{-1}.

κ-Car-(*R*,*R*)-**2**

κ-Car-(*R*,*R*)-**2** was prepared in a similar manner to *κ*-Car-(*S*)-**1** using (*R*,*R*)-**2** (962 mg, 3.40 mmol). Yield: 68%. ^{1}H NMR (400 MHz, D_2O) δ 7.86 (s, 5H), 5.53 (s, 5H), 5.26 (s, 5H), 5.05 (s, 14H), 4.94 (s, 2H), 4.39 (s, 2H), 4.23 (s, 24H), 4.07 (s, 7H), 3.40 (s, 2H), 3.11 (s, 4H), 1.60 (s, 41H), 1.35 (s, 4H). IR (ATR): 3370, 2956, 2337, 1647, 1374, 1214, 1158, 1124, 1034, 919, 844, 700, 602, 548, 526, 513 cm^{-1}.

κ-Car-(*S*)-**1** (5 equiv.)

κ-Car-(*S*)-**1** was prepared in a similar manner to *κ*-Car-(*S*)-**1** using 5 equiv. of (*S*)-**1** (433 mg, 1.70 mmol). Yield: 65%.

κ-Car-(*S*)-**1** (3 equiv.)

κ-Car-(*S*)-**1** was prepared in a similar manner to *κ*-Car-(*S*)-**1** using 3 equiv. of (*S*)-**1** (260 mg, 1.02 mmol). Yield: 83%.

Asymmetric Friedel‑Crafts alkylation[S2)]

The asymmetric Friedel‑Crafts reaction was carried out according to the literature as shown in Scheme 3.[S2)] Besides, the enantiomeric excess (*ee*) of the product, (*R*)-3-(1-methyl-1H-indol-3-yl)butanal, was determined by HPLC using a DAICEL CHIRALPAK AD-3 (eluent: hexane/EtOH = 98/2 (v/v)), 1 mL/min) after the reduction to the corresponding alcohol using $NaBH_4$.[S2)]

(*S*)-**1** (Table 1, entry 1)

In a 10 mL eggplant flask, (*S*)-**1** (0.100 mmol, 25.5 mg) was charged with CH_2Cl_2 (0.850 mL), isopropanol (0.150 mL), and trifluoroacetic acid (TFA, 0.100 mmol, 7.7 μL), then stirred at 0 °C for 5 min. Croton aldehyde (125 μL, 1.50 mmol) was added to the solution, and after stirring for additional 10 min, 1-methylindole (64.0 μL, 0.500 mmol) was added. After stirring for 90 minutes, the reaction mixture was passed through silica gel plug with Et_2O and then concentrated. The resulting residue was purified by silica gel

chromatography (eluent: toluene) to afford (*R*)-3-(1-methyl-1H-indol-3-yl)butanal **3a** (94.1 mg) in 94% isolated yield. The enantioselectivity was determined by subjecting approximately 10 mg (0.049 mmol) of the (*R*)-3-(1-methyl-1H-indol-3-yl)butanal to an excess amount of $NaBH_4$ (18.6 mg, 0.490 mmol) and EtOH (1 mL). After 15 min, the solution was treated with saturated aqueous $NaHCO_3$, and the mixture was extracted with CH_2Cl_2. ^{1}H NMR[S2)] (400 MHz, $CDCl_3$) δ 9.74 (d, J = 1.8 Hz, 1H), 7.63–7.53 (m, 1H), 7.30–7.21 (m, 2H), 7.13–7.05 (m, 1H), 6.83 (s, 1H), 3.74 (d, J = 1.4 Hz, 3H), 2.95–2.83 (m, 1H), 2.73–2.67 (m, 1H), 1.42 (d, J = 6.9 Hz, 3H). *Ee:* 36% (*R*), *S*-isomer: t_r = 23.5 min and *R*-isomer t_r = 25.6 min.

(*R*)-**1** (Table 1, entry 2)

(*R*)-**1** (0.100 mmol, 25.5 mg) was used as a catalyst. NMR yield: 98%, isolated yield: 88%. *Ee:* 35% (*S*).

Alg-(*S*)-**1** (Table 1, entry 3)

Alg-(*S*)-**1** (0.100 mmol, 43.0 mg) was used as a catalysts. NMR yield: 89%. *Ee:* 21% (*R*).

Alg-(*R*)-**1** (Table 1, entry 4)

Alg-(*R*)-**1** (0.100 mmol, 43.0 mg) was used as a catalyst. NMR yield: 99%. *Ee:* 42% (*S*).

Alg-(*R*)-$\mathbf{1}_{2nd}$ (Table 1, entry 6)

Alg-(*R*)-$\mathbf{1}_{2nd}$, which was collected by filtration from the reaction mixture using Alg-(*R*)-**1**, was reused again. NMR yield: 96%. *Ee:* 17% (*S*).

Alg-(*R*)-**1** (10 mol%) (Table 1, entry 7)

The catalytic amount of Alg-(*R*)-**1** (0.050 mmol, 21.5 mg) was reduced to 10 mol% and used for the same reaction. NMR yield: 91%. *Ee:* 10% (*S*).

κ-Car-(*R*,*R*)-$\mathbf{2}_{2nd}$ (Table 2, entry 9)

κ-Car-(*R*,*R*)-**2** (226 mg) which was recovered from 1st cycle (Table 2 entry 8) was used as a catalyst. NMR yield: 99%. *Ee:* 84% (*S*).

κ-Car-(*R*,*R*)-$\mathbf{2}_{3rd}$ (Table 2, entry 10)

κ-Car-(*R*,*R*)-**2** (183 mg) which was recovered from 2nd cycle (Table 2 entry 9) was used as catalyst. NMR yield: 96%. *Ee:* 67% (*S*).

κ-Car (Table 2, entry 11)

κ-Car (0.100 mmol, 76.4 mg) was used as a catalyst. NMR yield: 98%. *Ee:* 0%.

(*R*,*R*)-**2** (1 mol%) (Table S1, entry 1)

(*R*,*R*)-**2** (1.2 mg, 5.0×10^{-3} mmol,) was used as a catalyst. NMR yield: 77%. *Ee:* 85% (*S*).

(*R*,*R*)-**2** (5 mol%) (Table S1, entry 2)

(*R*,*R*)-**2** (6.2 mg, 0.0250 mmol,) was used as a catalyst. NMR yield: 99%. *Ee:* 86% (*S*).

(*R*,*R*)-**2** (10 mol%) (Table S1, entry 3)

(*R*,*R*)-**2** (12.3 mg, 0.0500 mmol,) was used as a catalyst. NMR yield: 99%. *Ee:* 86% (*S*).

κ-Car-(*R*,*R*)-**2** (5 mol%) (Table S1, entry 5)

κ-Car-(*R*,*R*)-**2** (41.9 mg) was used as a catalyst. NMR yield: 94%. *Ee:* 84% (*S*).

κ-Car-(*R*,*R*)-**2** (10 mol%) (Table S1, entry 6)

κ-Car-(*R*,*R*)-**2** (83.7 mg) was used as a catalyst. NMR yield: 98%. *Ee:* 86% (*S*).

κ-Car-(*R*,*R*)-**2** (r.t., 60 min) (Table S2, entry 1)

κ-Car-(*R*,*R*)-**2** (81.0 mg) was used as catalyst, and the reaction time was 1 hours and reaction in room temperature. NMR yield: 99%. *Ee:* 74% (*S*).

κ-Car-(*R*,*R*)-**2** (–50 °C, 180 min) (Table S2, entry 3)

κ-Car-(*R*,*R*)-**2** (106 mg) was used as a catalyst, and the reaction temperature was –50 °C and the reaction time was 3 hours. NMR yield: 98%. *Ee:* 86% (*S*).

^{1}H NMR spectra

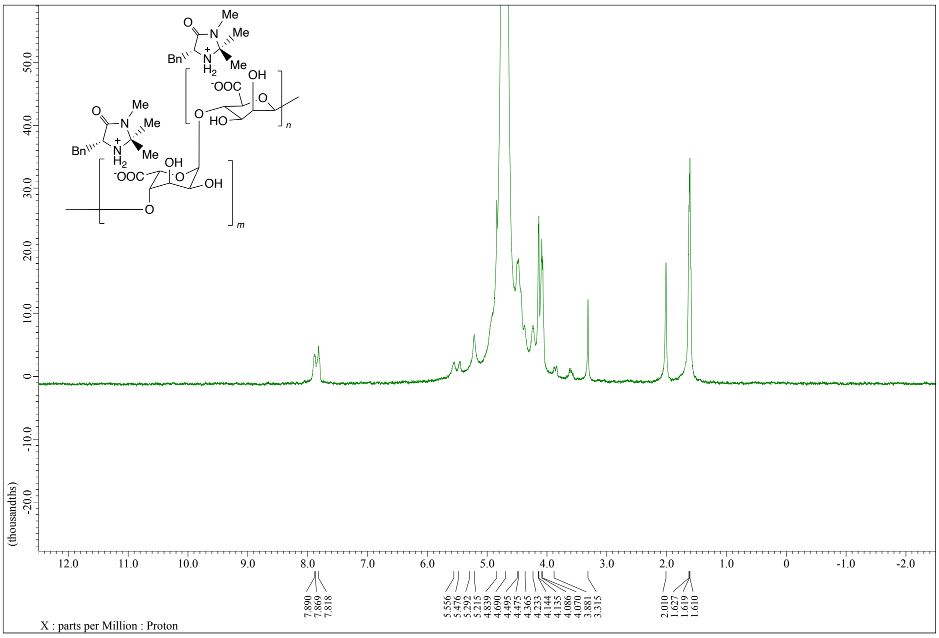

Fig. S1 ^{1}H NMR spectrum of Alg-(*R*)-**1** measured at 400 MHz in D_2O at 80 °C.

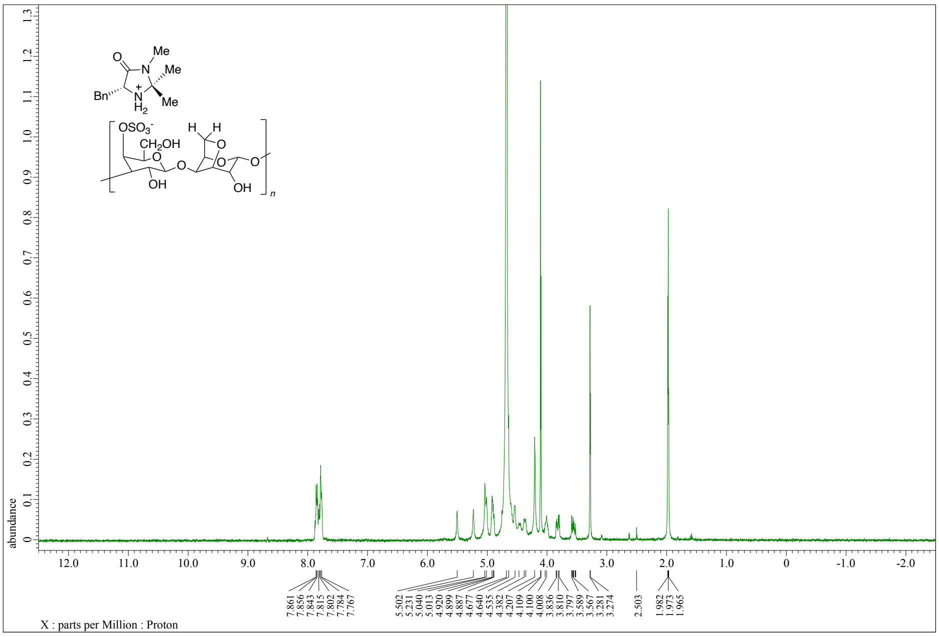

Fig. S2 ^{1}H NMR spectrum of *κ*-Car-(*R*)-**1** measured at 400 MHz in D_2O at 80 °C.

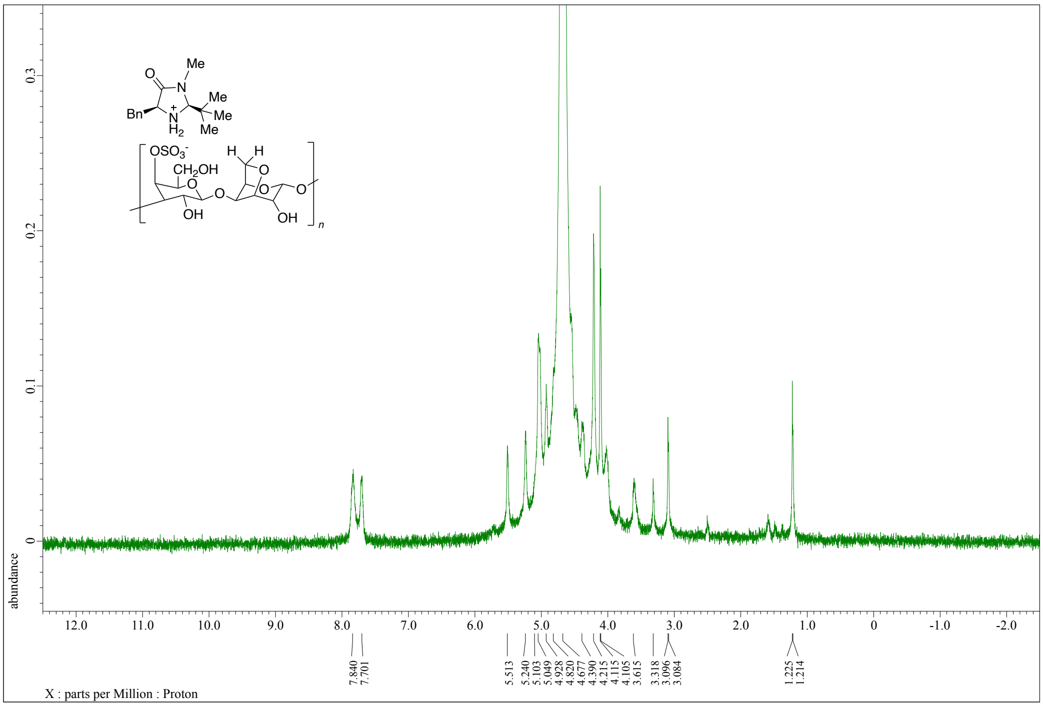

Fig. S3 ^{1}H NMR spectrum of *κ*-Car-(*S*,*S*)-**2** measured at 400 MHz in D_2O at 80 °C.

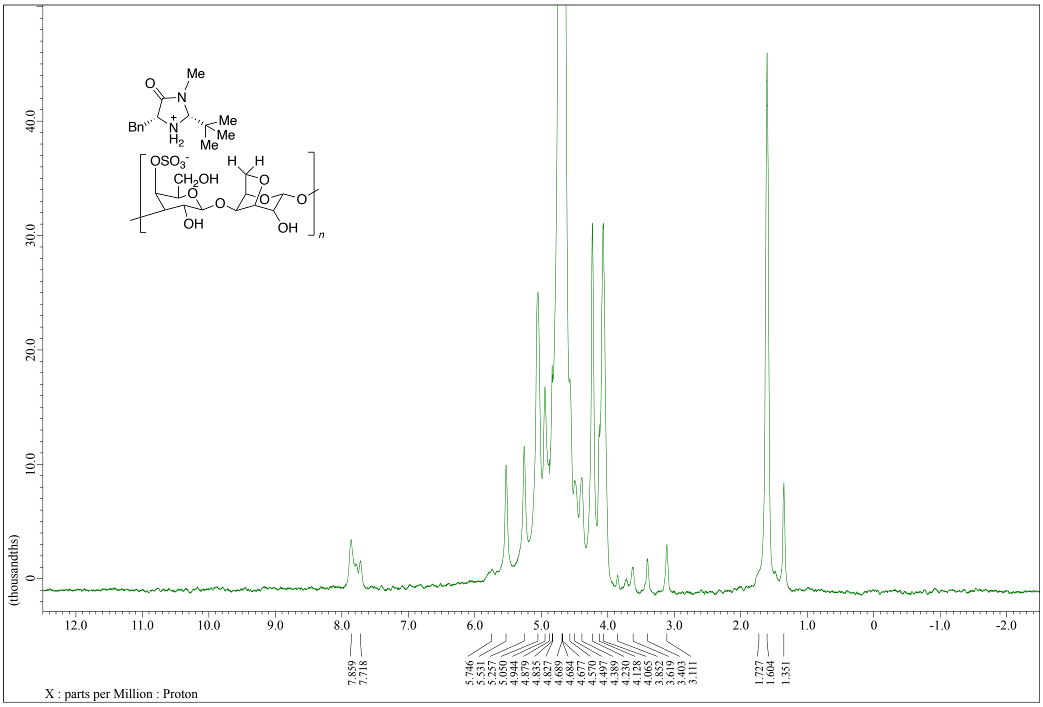

Fig. S4 ^{1}H NMR spectrum of *κ*-Car-(*R*,*R*)-**2** measured at 400 MHz in D_2O at 80 °C.

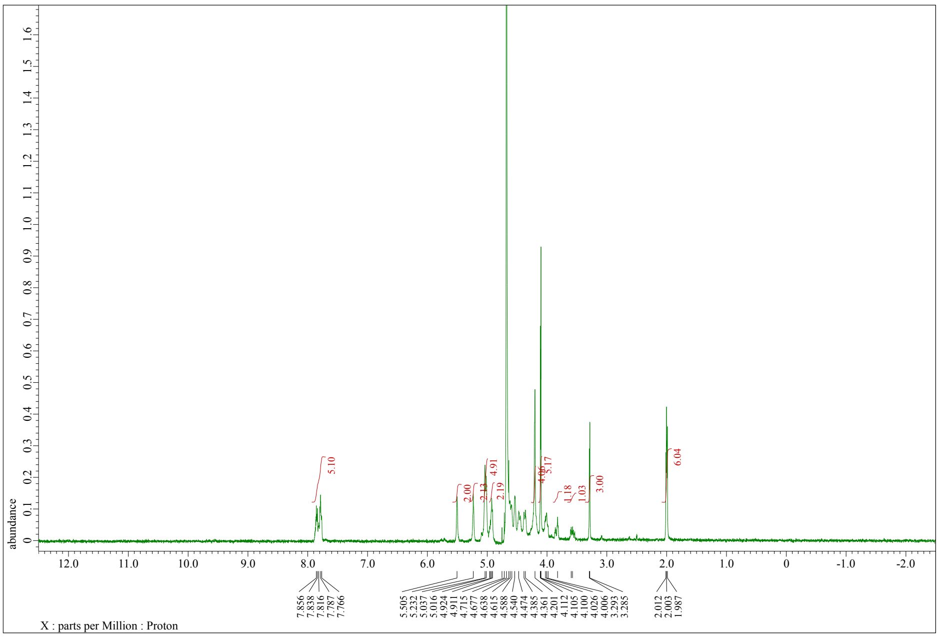

Fig. S5 ^{1}H NMR spectrum of *κ*-Car-(*S*)-**1** (5 equiv.) measured at 400 MHz in D_2O at 80 °C.

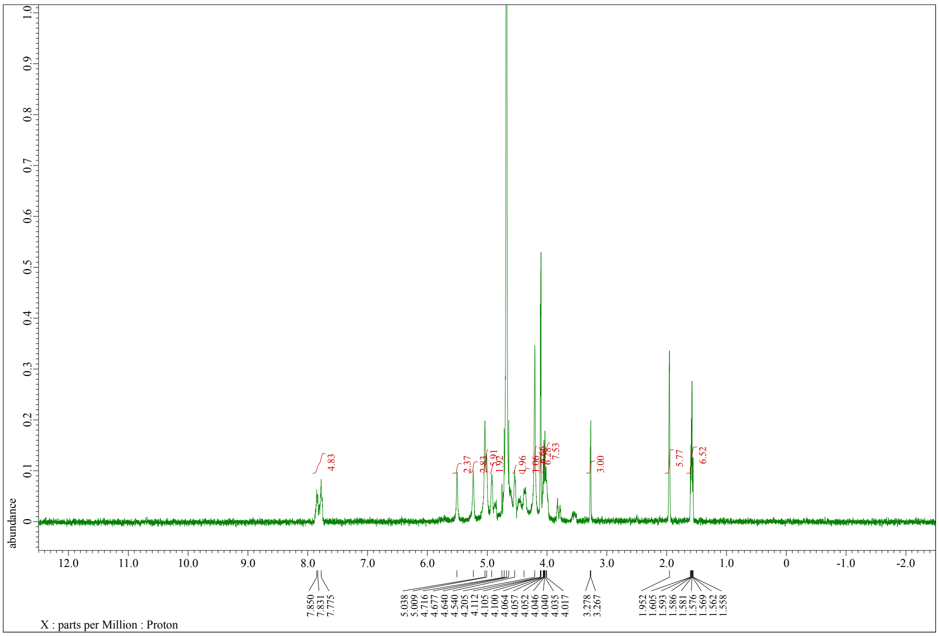

Fig. S6 ^{1}H NMR spectrum of *κ*-Car-(*S*)-**1** (3 equiv.) measured at 400 MHz in D_2O at 80 °C.

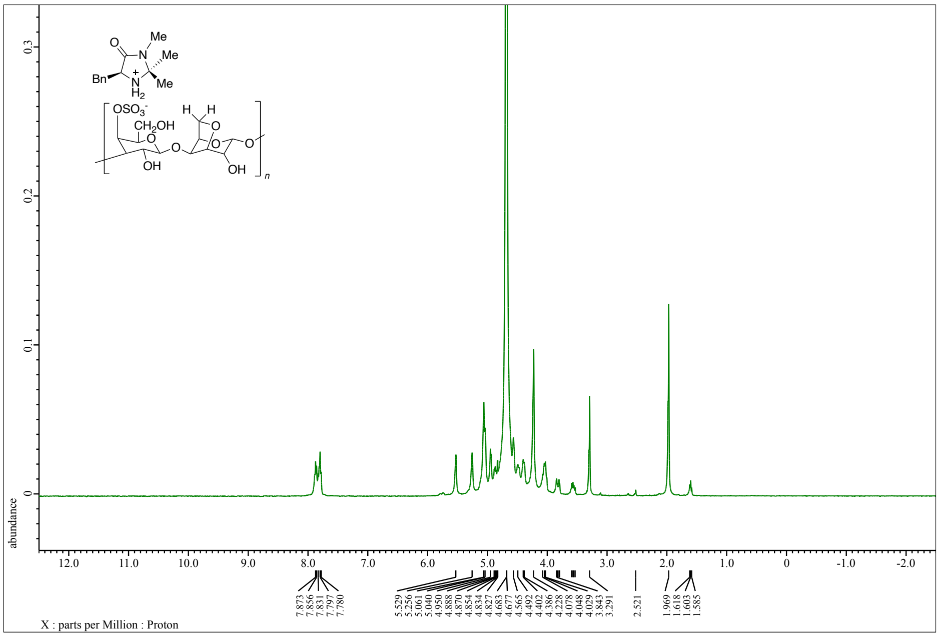

Fig. S7 ^{1}H NMR spectrum of *κ*-Car-(*S*)-**1** obtained without using 15-crown-5 measured at 400 MHz in D_2O at 80 °C.

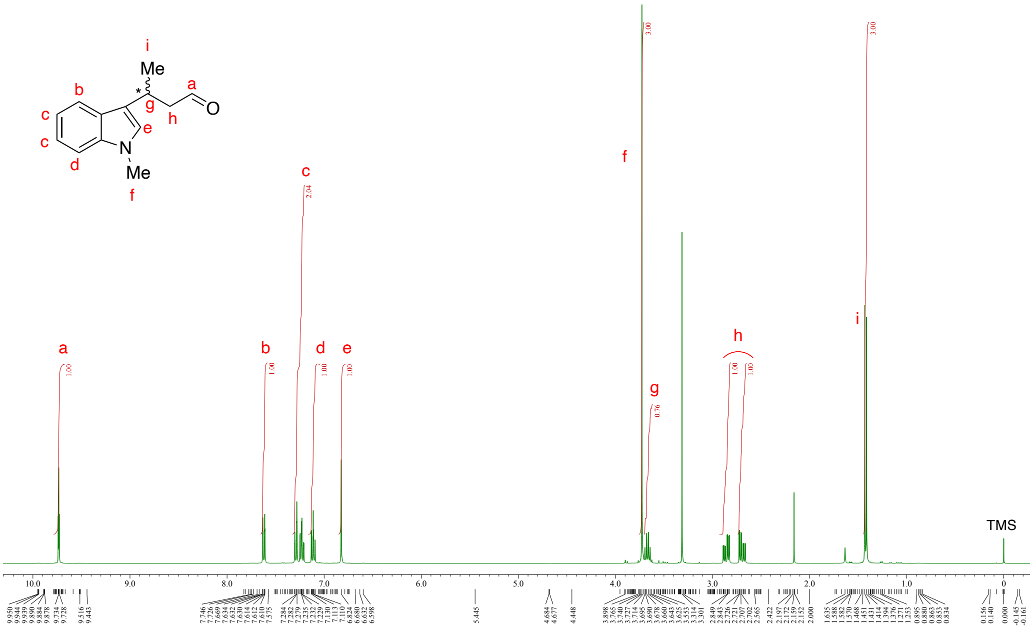

Fig. S8 ^{1}H NMR spectrum of **3a** at measured at 400 MHz in $CDCl_3$.

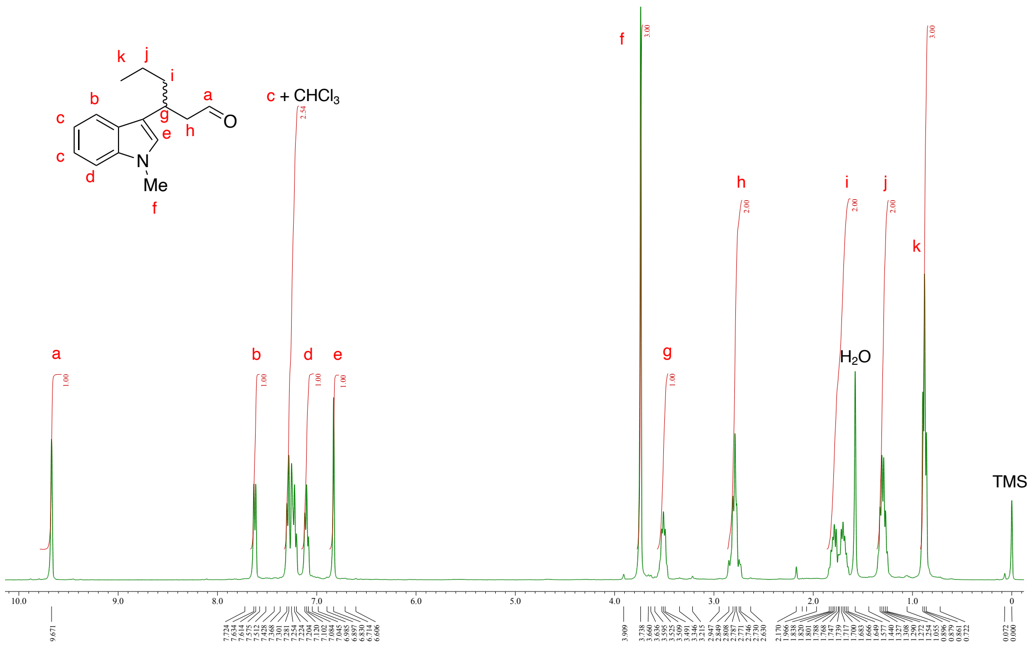

Fig. S9 ^{1}H NMR spectrum of **3b** at measured at 400 MHz in $CDCl_3$.

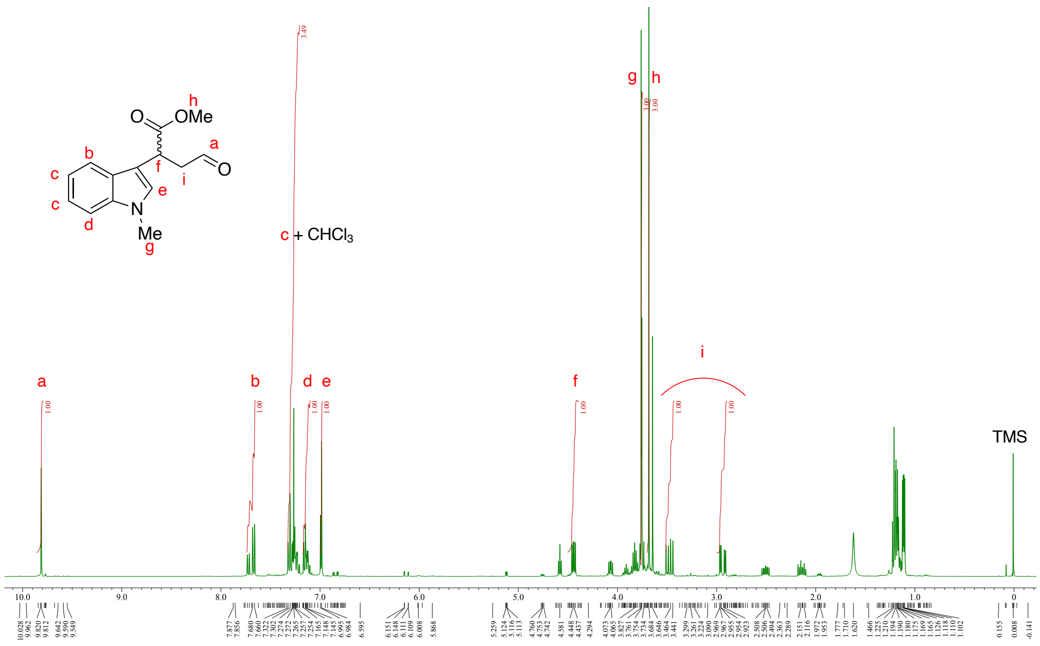

Fig. S10 ^{1}H NMR spectrum of **3c** measured at 400 MHz in $CDCl_3$.

ATR–IR spectra

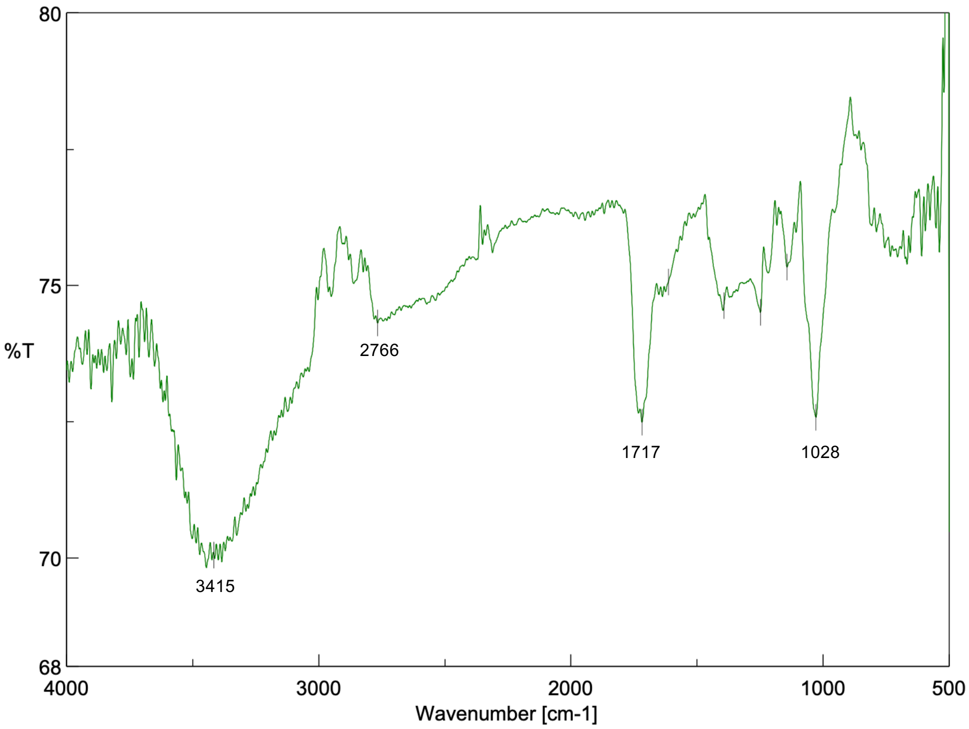

Fig. S11 ATR–IR spectrum of Alg-(*R*)-**1**.

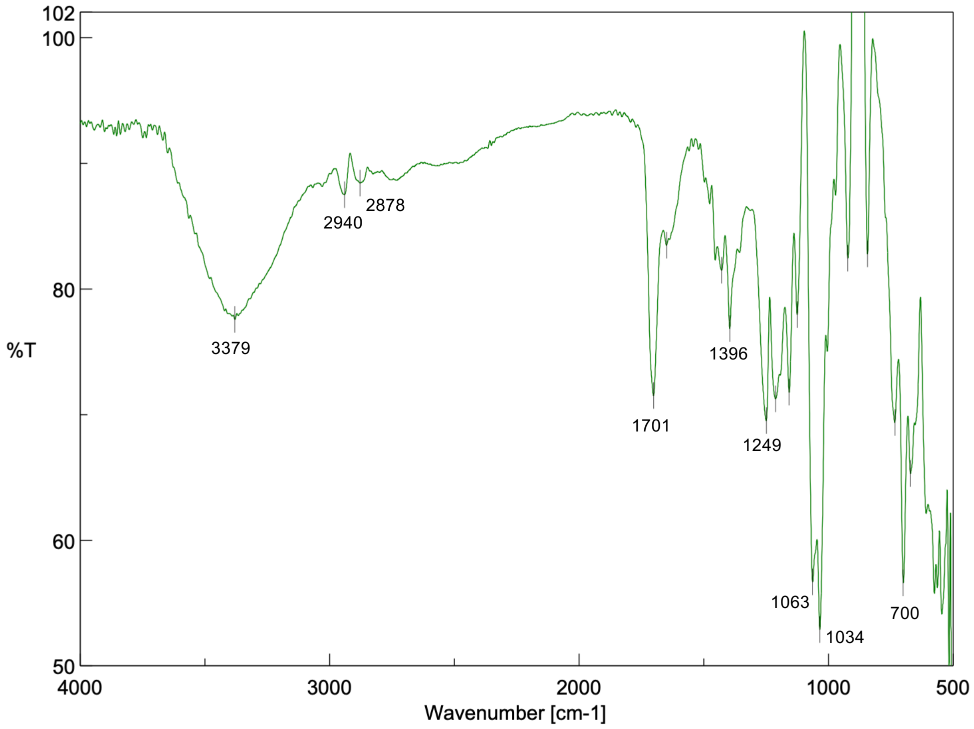

Fig. S12 ATR–IR spectrum of *κ*-Car-(*R*)-**1**.

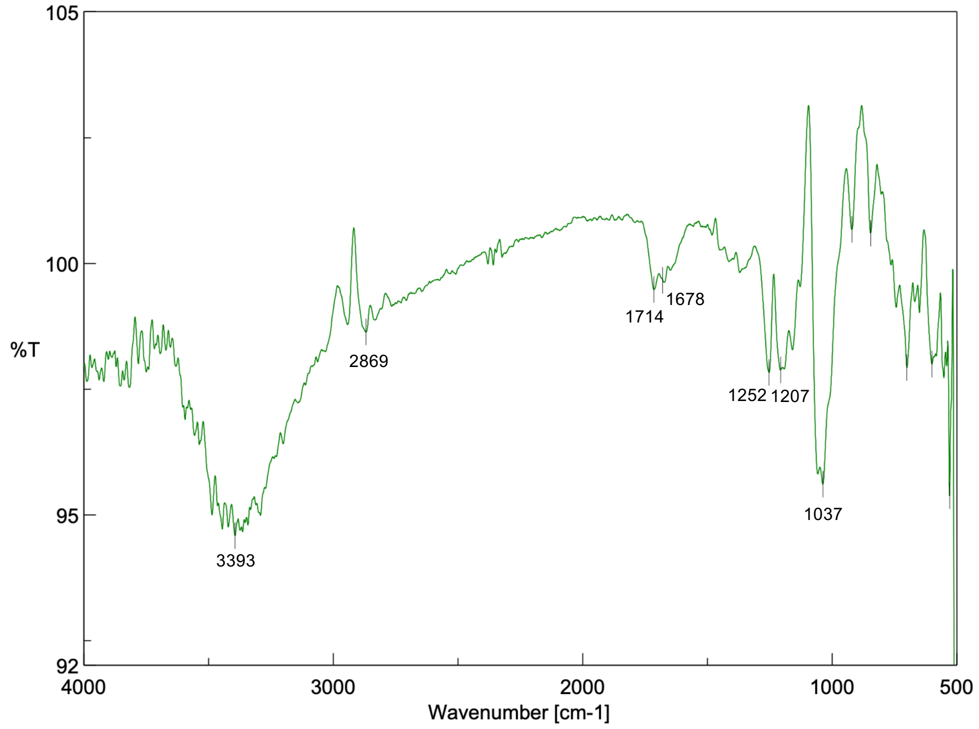

Fig. S13 ATR–IR spectrum of *κ*-Car-(*S*,*S*)-**2**.

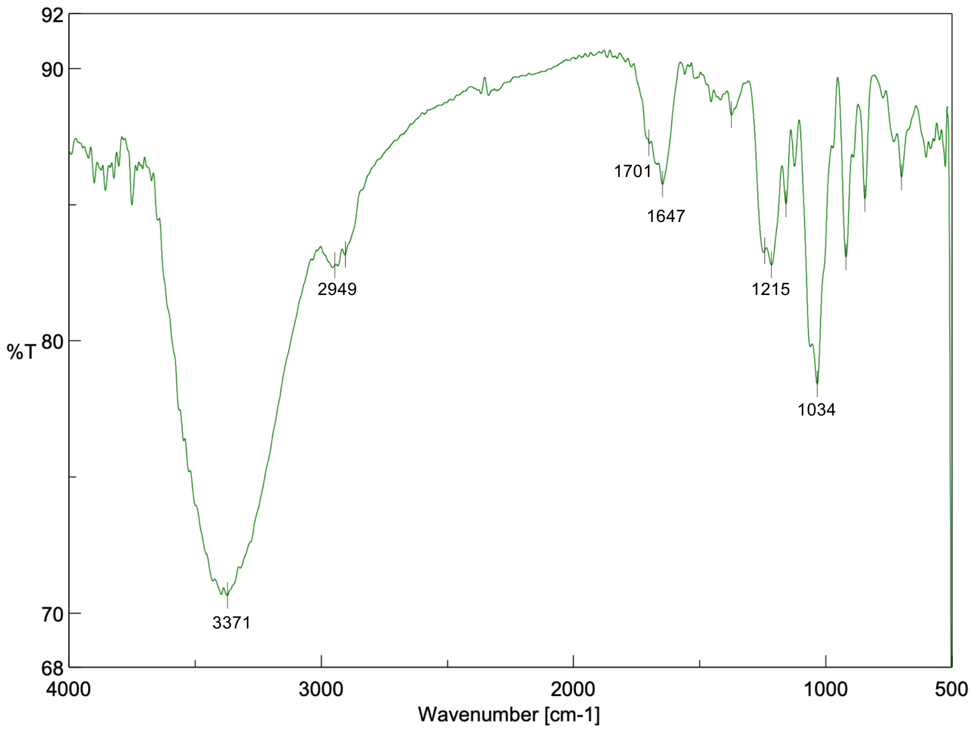

Fig. S14 ATR–IR spectrum of *κ*-Car-(*R*,*R*)-**2**.

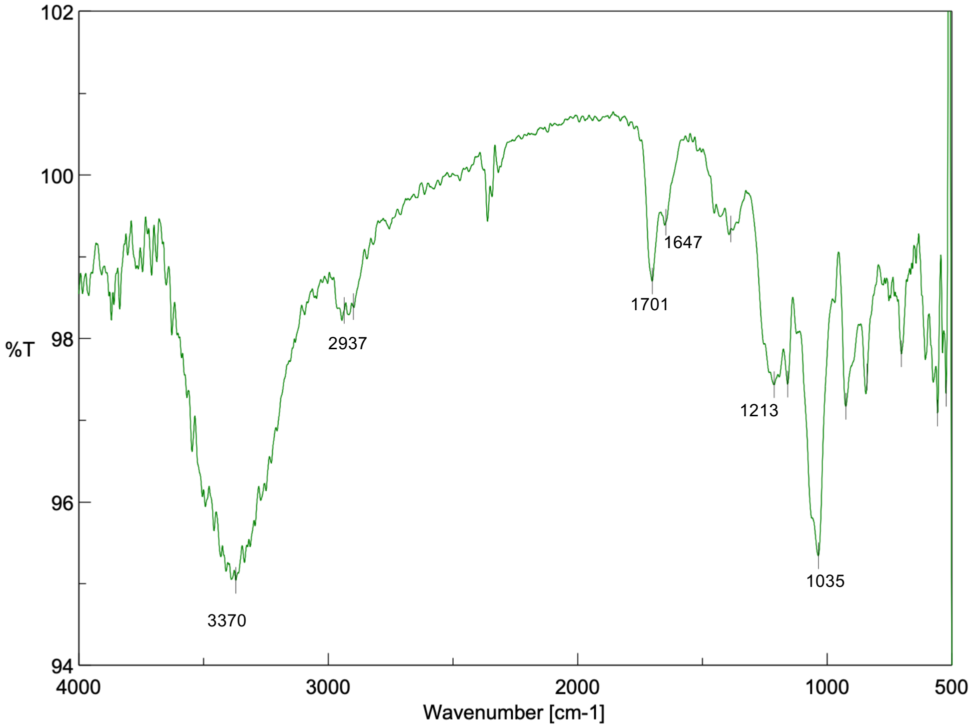

Fig. S15 ATR–IR spectrum of *κ*-Car-(*S*)-**1** (5 equiv.).

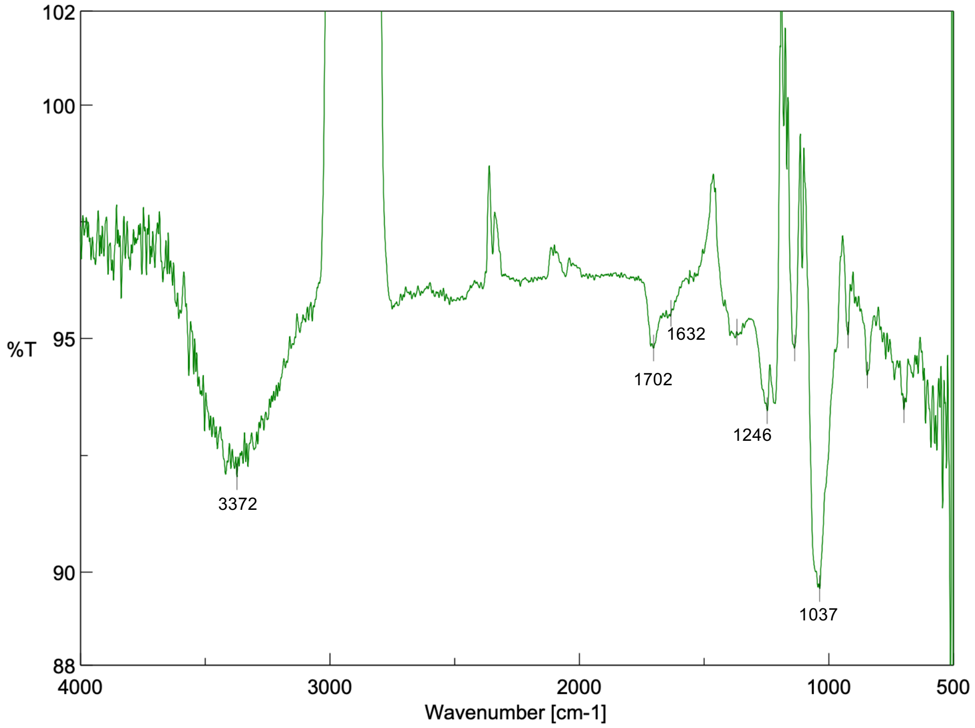

Fig. S16 ATR–IR spectrum of *κ*-Car-(*S*)-**1** (3 equiv.).

UV–vis diffuse reflectance spectra

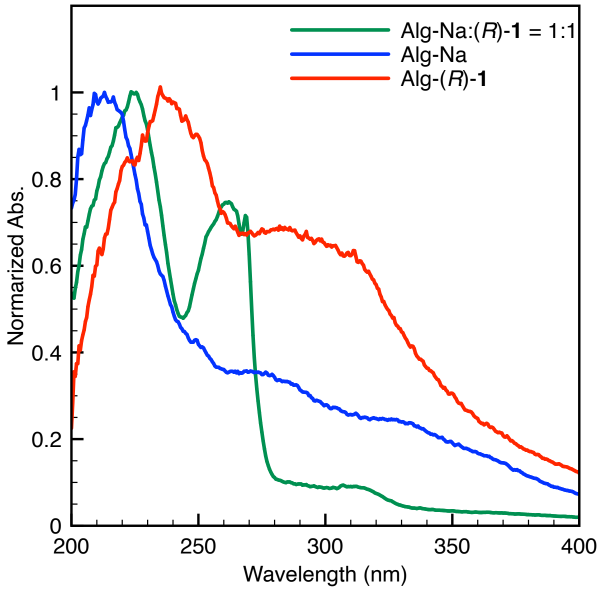

Fig. S17 UV–vis diffuse reflectance spectra of Alg-Na, Alg-(*R*)-**1** and Alg-Na/(*R*)-**1** = 1/1 (unit molar ratio). Note the spectra were measured because Alg-(*R*)-**1** was partly insoluble in various solvents including H_2O as shown in Fig. S18.

UV–vis absorption spectra in solution

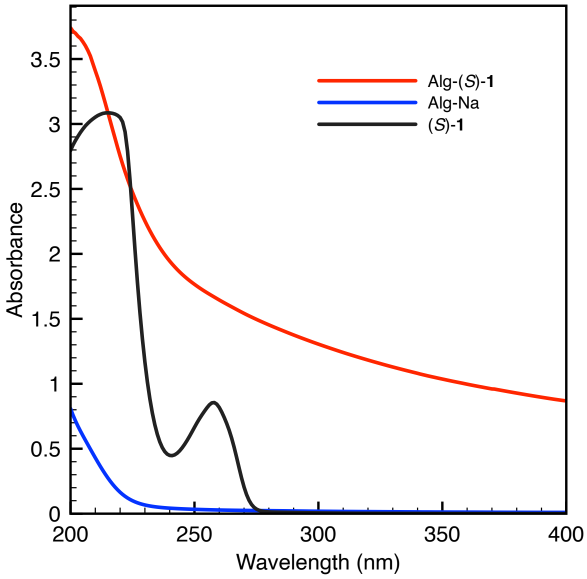

Fig. S18 UV–vis absorption spectra of Alg-Na, (*S*)-**1** and Alg-(*S*)-**1** measured in H_2O (c = 5.0 mM).

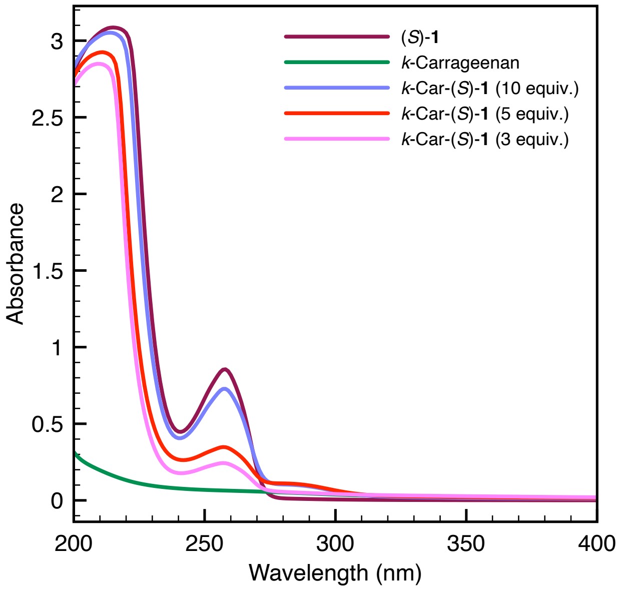

Fig. S19 UV–vis absorption spectra of *κ*-Car, (*S*)-**1**, *κ*-Car-(*S*)-**1** (10 equiv.), *κ*-Car-(*S*)-**1** (5 equiv.) and *κ*-Car-(*S*)-**1** (3 equiv.) measured in H_2O (c = 5.0 mM).

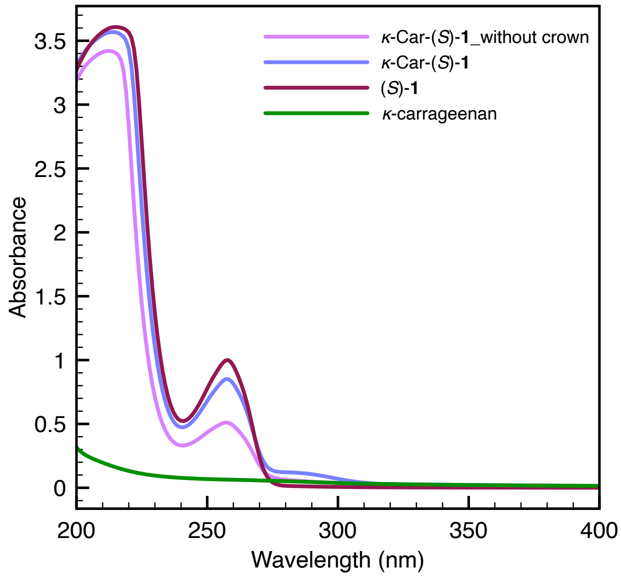

Fig. S20 UV–vis absorption spectra of *κ*-Car, (*S*)-**1**, *κ*-Car-(*S*)-**1** and *κ*-Car-(*S*)-**1** obtained without using 15-crown-5 measured in H_2O (c = 5.0 mM).

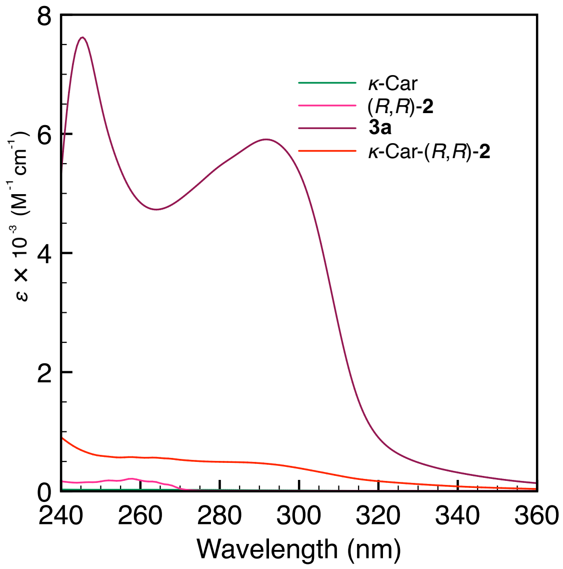

Fig. S21 UV–vis absorption spectra of *κ*-Car, (*R*,*R*)-**2**, and *κ*-Car-(*R*,*R*)-**2** (after 3rd cycle) measured in H_2O, **3a** measured in $CHCl_3$ (c = 0.5 mM).

CD spectroscopic analysis and density functional theory (DFT) calculation

CD and UV–vis absorption spectra of *κ*-Car, (*S*)-/(*R*)-**1**, (*S*,*S*)-/(*R*,*R*)-**2**, *κ*-Car-(*S*)-/(*R*)-**1** and *κ*-Car-(*S*,*S*)-/(*R*,*R*)-**2** were measured in H_2O to gain information of chirality of the *κ*-Car-supported catalysts (Fig. S22 and S23). (*S*)-**1** and *κ*-Car-(*S*)-**1** exhibited a positive CD peak at 200–220 nm, whereas (*R*)-**1** and *κ*-Car-(*R*)-**1** showed a negative peak at the same region, indicating that the point chirality of (*R*)-**1** unit dominantly determined the sign of the CD peak at this region. The CD intensity of *κ*-Car-(*R*)-**1** was stronger than that of (*R*)-**1**, while the CD intensity of *κ*-Car-(*S*)-**1** was weaker than that of (*S*)-**1**. These results suggest that *κ*-Car-(*R*)-**1** possesses more chirally fixed environment than (*R*)-**1** by the cooperative interaction between (*R*)-**1** and *κ*-Car, enhancing the enantioselectivity in the Friedel-Crafts reaction, while *κ*-Car-(*S*)-**1** possesses less chirally fixed environment than (*S*)-**1**.

To confirm this tendency, density functional theory (DFT) calculations were conducted on the model compounds for *κ*-Car-(*S*)-/(*R*)-**1**. *i*-Car double helix determined by X-ray diffraction was used as the initial geometry of *κ*-Car model.[S3,S4)] As *i*-Car has additional $–OSO_3H$ group compared to *κ*-Car, it was substituted with a hydrogen atom in a similar manner to the work by Sekkal-Rahal and coworkers.[S5)] The X-ray crystallographic data was used for the initial geometries of (*S*)-**1** by removing the chloride anion.[S6)] The geometries of (*R*)-**1** were obtained by inverting the geometry optimized conformer of (*S*)-**1**. After optimizing the geometries, time-dependent DFT calculations (TD-DFT) were further conducted to simulate the CD and UV–vis absorption spectra. Fig. S24 shows the simulated CD and UV–vis spectra of *κ*-Car-model, (*S*)-/(*R*)-**1** and *κ*-Car-(*S*)-/(*R*)-**1**-models. The absorption maxima were simulated at shorter wavelength than the observed ones, which often occurs upon employing ωB97X-D functional.[S7)] The signs of CD signals of the *S* and *R* enantiomers matched the observed ones. Moreover, the CD intensity of *κ*-Car-(*R*)-**1**-model was almost twice larger than that of (*R*)-**1**, whereas that of *κ*-Car-(*S*)-**1**-model was smaller than that of (*S*)-**1**, coincident with the experimentally observed tendency. Thus, the computational simulation was valuable to understand the enantioselectivity of the *κ*-Car-supported catalysts. Meanwhile, *κ*-Car-(*S*,*S*)-/(*R*,*R*)-**2** did not show obvious CD intensity changes as shown in Fig. S23. The high enantioselectivity of *κ*-Car-(*S*,*S*)-/(*R*,*R*)-**2** should originate from the chirally ordered environment at the transition state of the reaction.

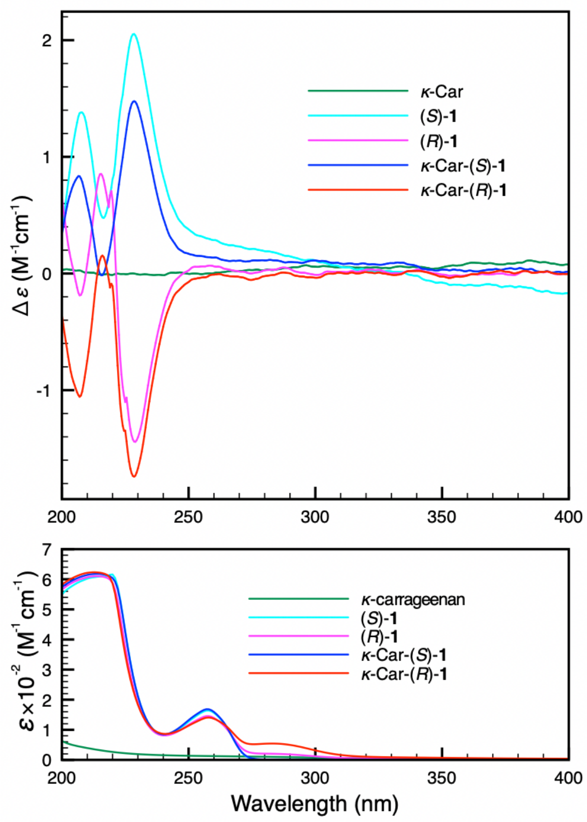

Fig. S22 CD and UV–vis absorption spectra of *κ*-Car, (*S*)-/(*R*)-**1**, *κ*-Car-(*S*)/(*R*)-**1** measured in H_2O at 22 °C (CD: c = 0.1 mM, UV: c = 5.0 mM).

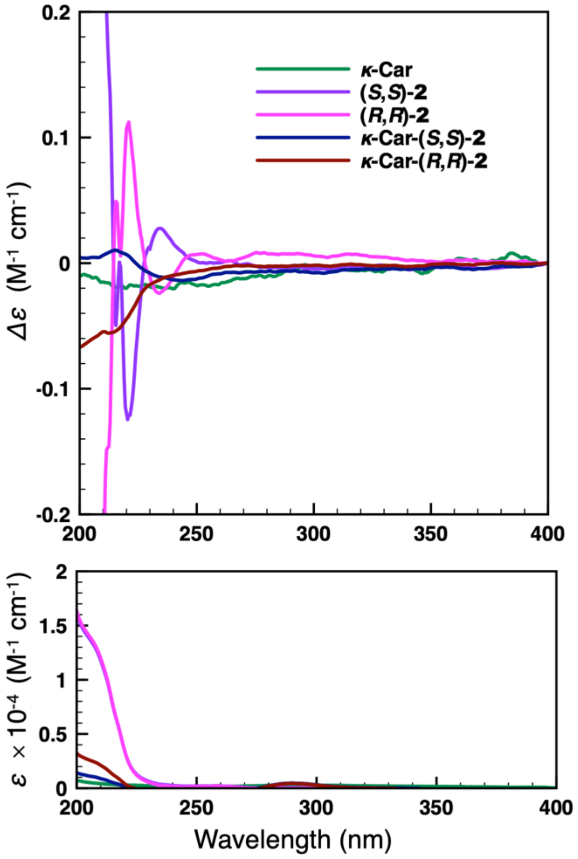

Fig. S23 CD and UV–vis absorption spectra of *κ*-Car, (*S*,*S*)-**2**, (*R*,*R*)-**2**, *κ*-Car-(*S*,*S*)-**2** and *κ*-Car-(*R,R*)-**2** measured in H_2O at 22 °C (CD: *c* = 0.1 mM, UV: *c* = 5.0 mM).

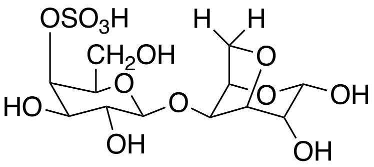

κ-Car-model

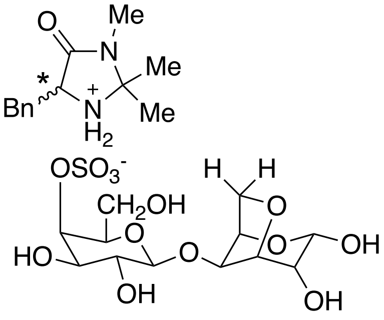

κ-Car-(*S*)-/(*R*)-**1**-model

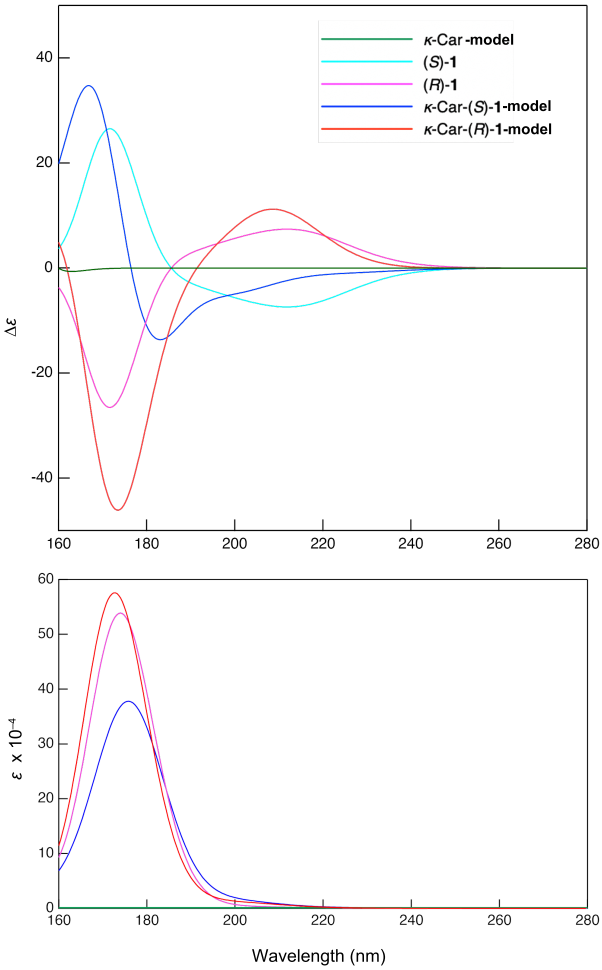

Fig. S24 Chemical structures of κ-Car-model and κ-Car-(*S*)-/(*R*)-**1**-model (top), and CD and UV–vis absorption spectra of κ-Car-model, (*S*)-**1**, (*R*)-**1**, κ-Car-(*S*)-**1**-model and κ-Car-(*R*)-**1**-model simulated by the TD-DFT method using ωB97X-D functional, with 6-31G* (C, H, N, O, S) basis sets, nstates = 40, plotted with peak half-width at half-height = 0.1 eV using GaussView 6 (bottom).

Friedel–Crafts Alkylation

Table S1. Effect of catalyst and cocatalyst loading on the Friedel-Crafts alkylation of 1-methylindole with crotonaldehyde using (*R*,*R*)-**2** and *κ*-Car-(*R*,*R*)-**2** [a)]

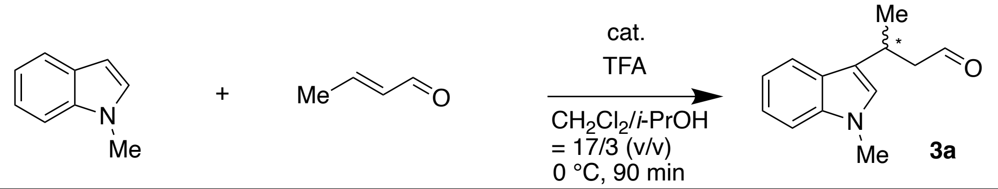

entry	cat.	catalyst and TFA (mol%) [b)]	yield (%) [c)]	*ee* (%) [d)]
1	(*R*,*R*)-**2**	1	77	85 (*S*)
2		5	99	86 (*S*)
3		10	99	86 (*S*)
4		20	96	78 (*S*)
5	*κ*-Car-(*R*,*R*)-**2**	5	94	84 (*S*)
6		10	98	86 (*S*)
7		20	99	85 (*S*)

[a)] Conditions: $[\text{1-methylindole}]_0$ = 0.5 M, $[\text{croton aldehyde}]_0$ = 1.5 M. [b)] The catalyst and TFA amounts vs 1-methylindole. [c)] Determined by ^{1}H NMR. [b)] Determined by HPLC using a DAICEL CHIRALPAK AD-3 eluted with hexane/EtOH = 98/2 (v/v) at a flow rate of 1.0 mL min^{-1}.

Table S2. Effect of temperature on the Friedel-Crafts alkylation of 1-methylindole with crotonaldehyde using *κ*-Car-(*R*,*R*)-**2** [a)]

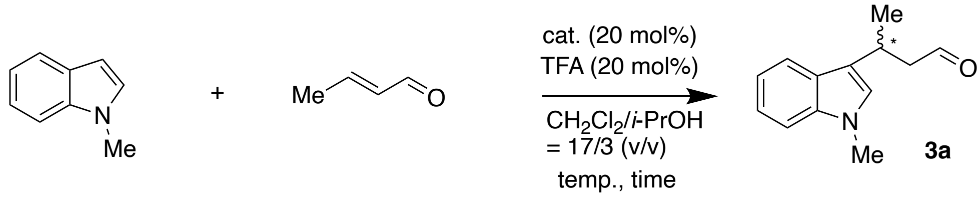

entry	temperature (°C)	time (min)	yield (%) [b)]	*ee* (%) [c)]
1	25	60	99	74 (*S*)
2	0	90	99	85 (*S*)
3	–50	180	98	86 (*S*)

[a)] Conditions: [1-methylindole]$_0$ = 0.5 M, [croton aldehyde]$_0$ = 1.5 M, [cat.] = [TFA] = 0.1 M in CH_2Cl_2/*i*-PrOH = 17/3 (v/v). [b)] Determined by ^{1}H NMR. [c)] Determined by HPLC using a DAICEL CHIRALPAK AD-3 eluted with hexane/EtOH = 98/2 (v/v) at a flow rate of 1.0 mL min^{-1}.

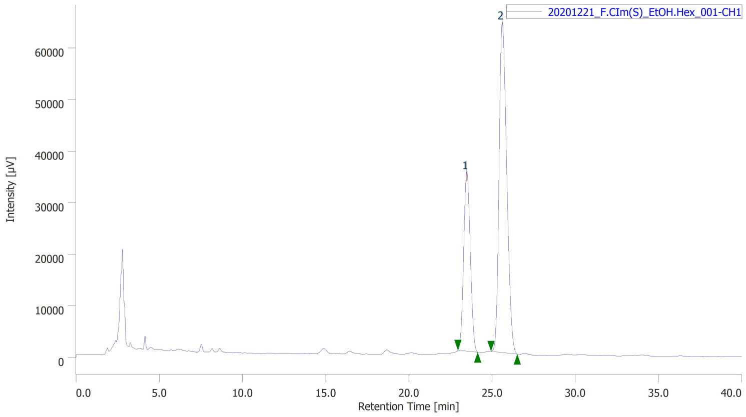

Fig. S25 HPLC chart of **3a** with (*S*)-**1** (Table 1, entry 1).

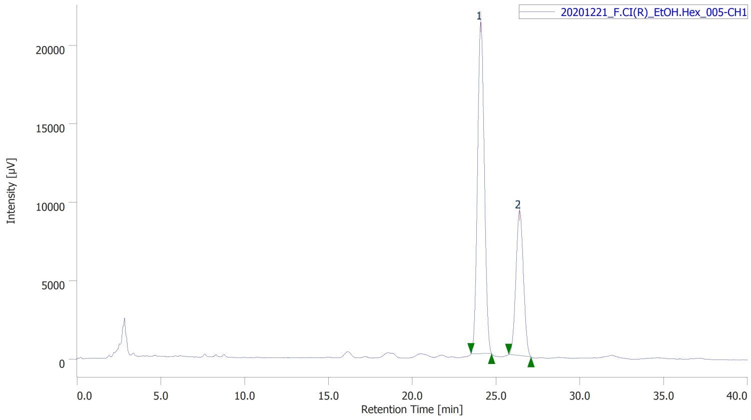

Fig. S26 HPLC chart of **3a** with (*R*)-**1** (Table 1, entry 2).

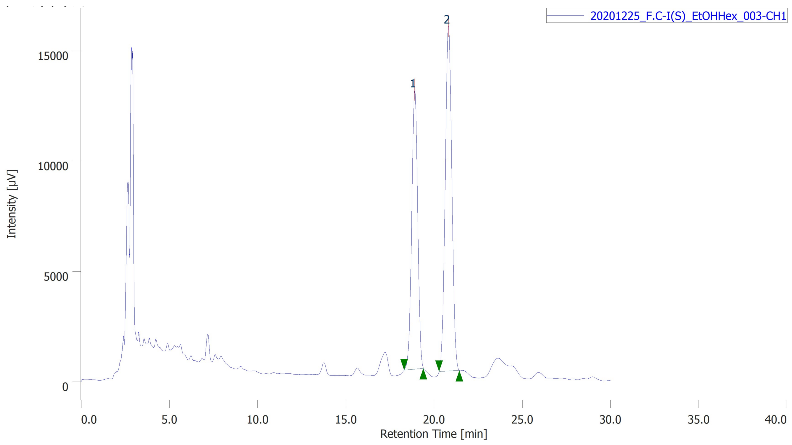

Fig. S27 HPLC chart of **3a** with Alg-(*S*)-**1** (Table 1, entry 3).

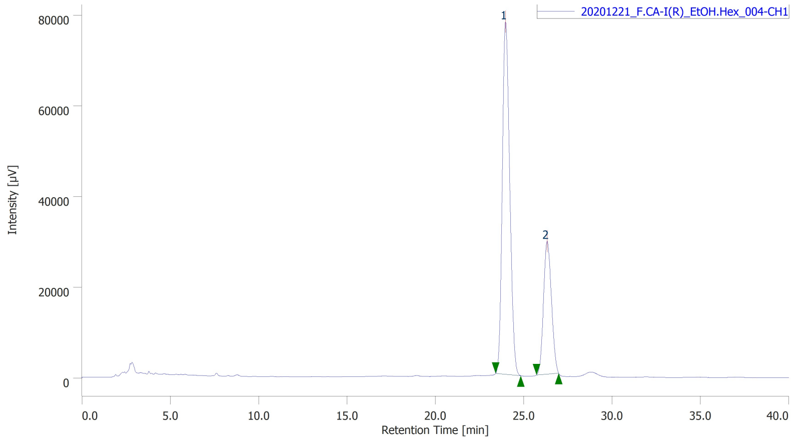

Fig. S28 HPLC chart of **3a** with Alg-(*R*)-**1** (Table 1, entry 4).

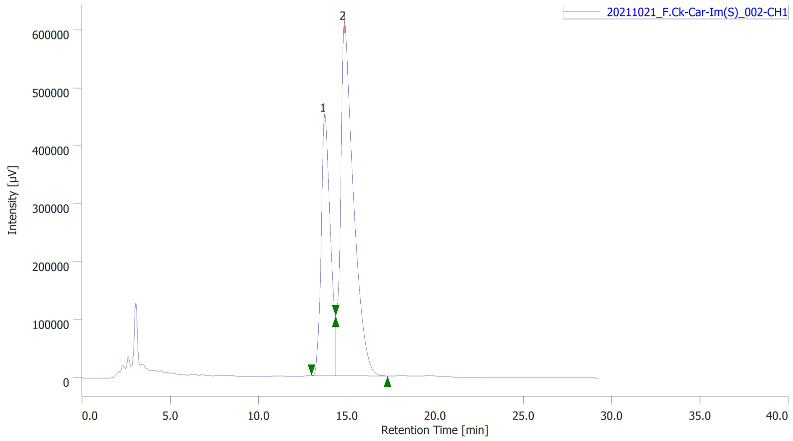

Fig. S29 HPLC chart of **3a** with *κ*-Car-(*S*)-**1** (Table 2, entry 3).

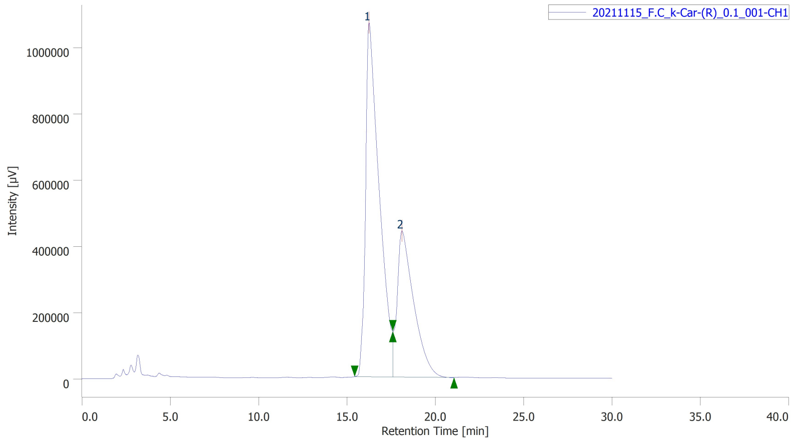

Fig. S30 HPLC chart of **3a** with *κ*-Car-(*R*)-**1** (Table 2, entry 4).

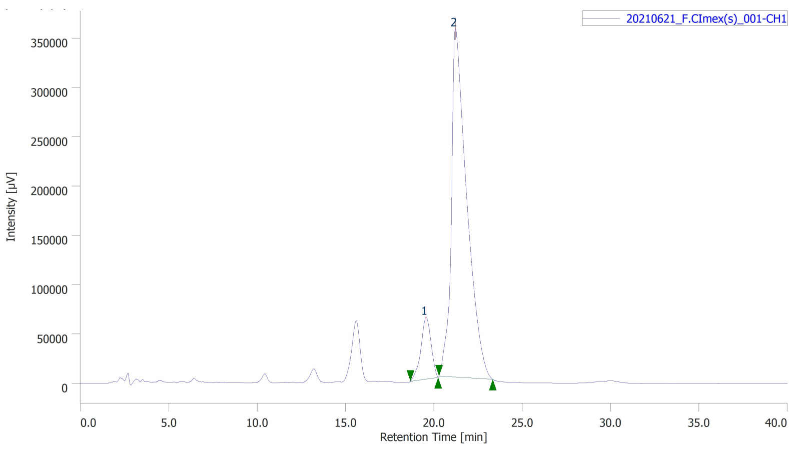

Fig. S31 HPLC chart of **3a** with (*S,S*)-**2** (Table 2, entry 5).

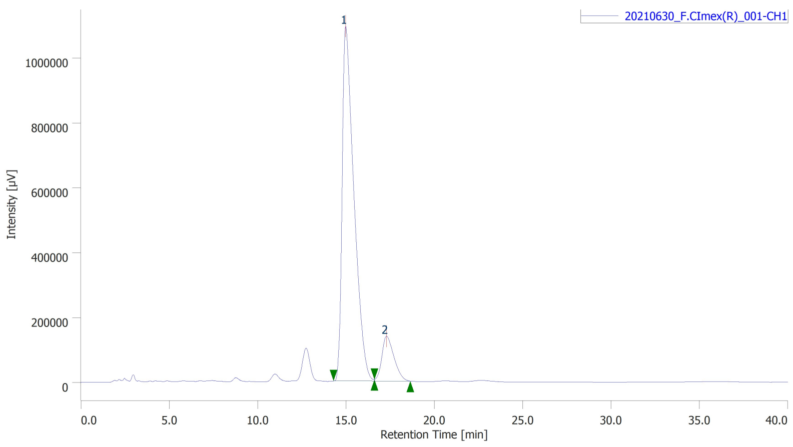

Fig. S32 HPLC chart of **3a** with (*R,R*)-**2** (Table 2, entry 6).

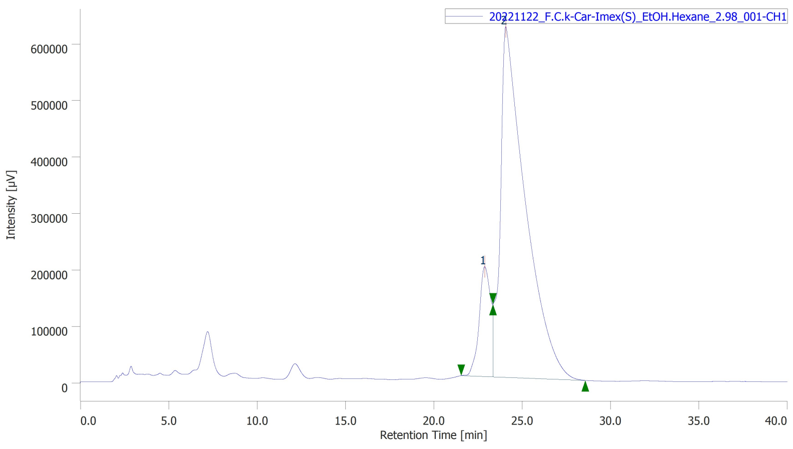

Fig. S33 HPLC chart of **3a** with *κ*-Car-(*S,S*)-**2** (Table 2, entry 7).

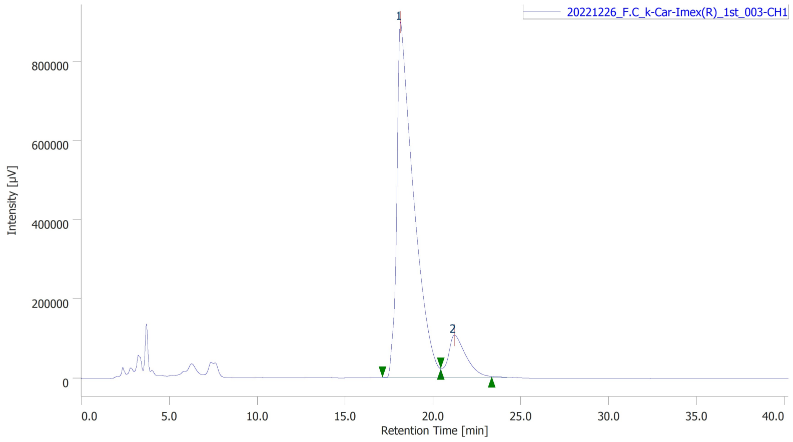

Fig. S34 HPLC chart of **3a** with *κ*-Car-(*R,R*)-**2** (Table 2, entry 8).

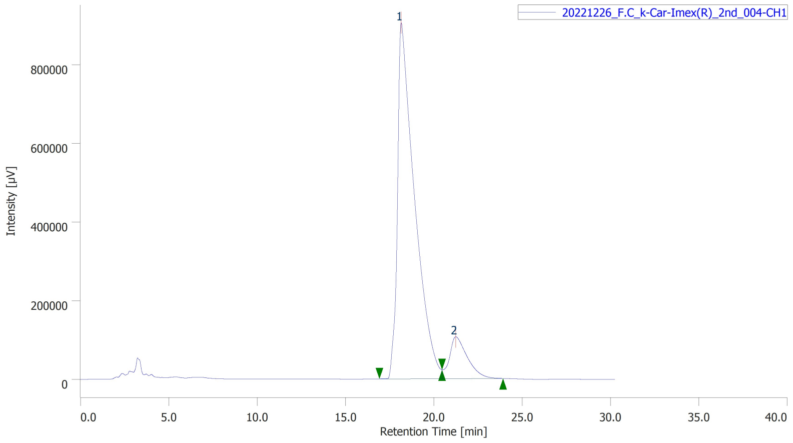

Fig. S35 HPLC chart of **3a** with *κ*-Car-(*R,R*)-**2** (Table 2, entry 9).

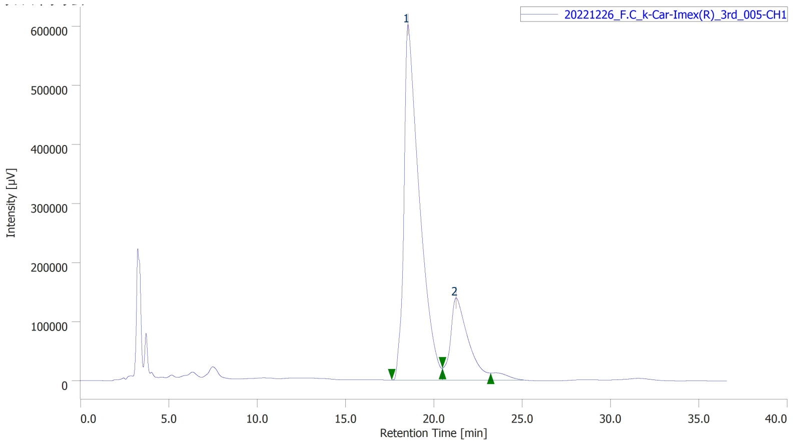

Fig. S36 HPLC chart of **3a** with *κ*-Car-(*R,R*)-**2** (Table 2, entry 10).

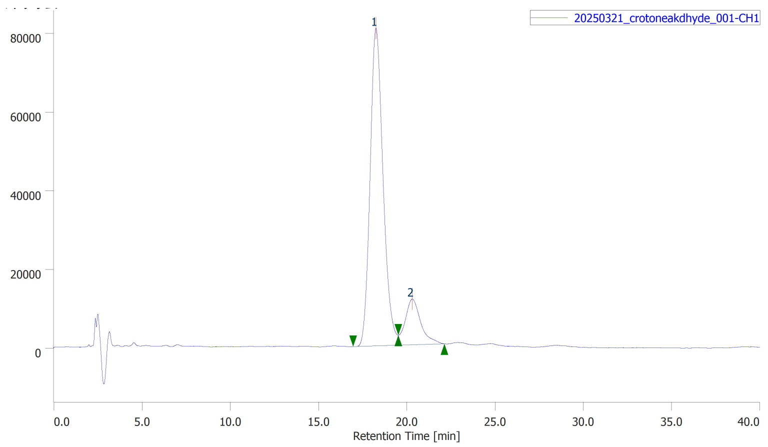

Fig. S37 HPLC chart of **3a** with *κ*-Car-(*R,R*)-**2** (Table 3, entry 1).

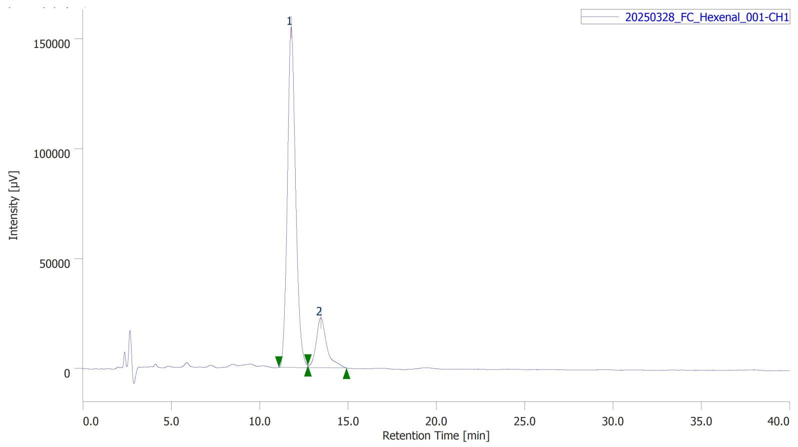

Fig. S38 HPLC chart of **3b** with *κ*-Car-(*R,R*)-**2** (Table 3, entry 2).

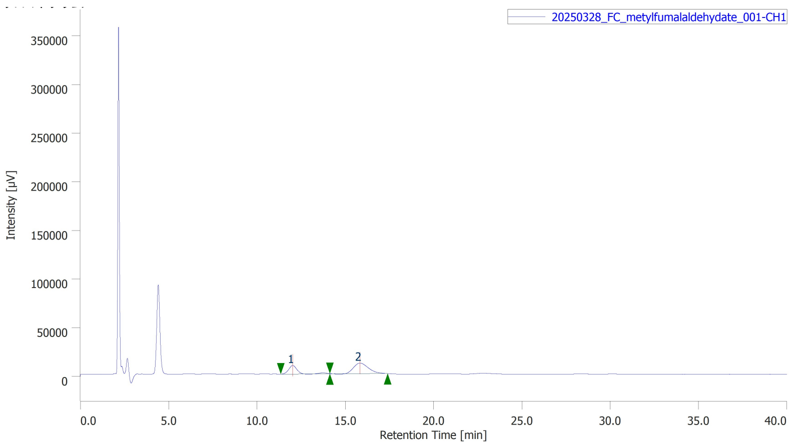

Fig. S39 HPLC chart of **3c** with *κ*-Car-(*R,R*)-**2** (Table 3, entry 3).

References

S1. M. Watanabe, T. Sakai, M. Oka, Y. Makinose, H. Miyazaki and H. Iida, *Adv. Synth. Catal.*, 2020, **362**, 255–260.

S2. F. Austin and D. W. C. MacMillan, *J. Am. Chem. Soc.,* 2002, **124**, 1172–1173.

S3. S. Arnott, W. Scott, D. A. Rees and C. G. A. McNab, *J. Mol. Biol.,* 1974, **90**, 257–267.

S4. S. Janaswarmy and R. Chandrasekaran, *Carbohydr. Res.*, 2002, **337**, 523–535.

S5. N. Yousfi, M. Sekkal-Rahal, A. Sayede and M. Springborg, *J. Comput. Chem.*, 2010, **31**, 1312–1320.

S6. J. C. Burley, R. Gilmour, T. J. Prior and G. M. Day, *Acta Cyst.*, 2008, **C64**, o10–o14.

S7. T. Sotani, T. Yajima, H. Sogawa and F. Sanda, *Macromolecules*, 2020, **53**, 11077–11088.
